# Neuronal MCT2 promotes angiogenesis via lactate in the developing mouse neocortex

**DOI:** 10.1038/s41418-025-01581-w

**Published:** 2025-10-04

**Authors:** Daehoon Lee, Anika Wu, Lingling Yao, Shreya Satish, Lin Mei, Wen-Cheng Xiong

**Affiliations:** 1 Department of Neurosciences, School of Medicine, Case Western Reserve University, Cleveland, OH USA; 2 Louis Stokes Cleveland Veterans Affairs Medical Center, Cleveland, OH USA

**Keywords:** Astrocyte, Neuroscience

## Abstract

Neural activity drives blood vessel (BV) formation and energy substrate delivery in the developing brain to meet rising metabolic demands; however, the underlying mechanisms remain poorly understood. In this study, we exposed neonatal mice to chronic whisker stimulation (WS), a paradigm known to enhance BV formation in the somatosensory (S1) cortex. Transcriptomic (RNA-seq) and spatial (RNA-scope) analyses revealed that WS upregulated monocarboxylate transporter 2 (MCT2) in cortical neurons and MCT1 in endothelial cells (ECs). These changes coincided with increased cortical lactate levels, elevated astrocytic vascular endothelial growth factor A (VEGFa), and enhanced angiogenesis. Functional experiments demonstrated that neuronal MCT2 is essential for mediating WS-induced angiogenic and metabolic responses. Mechanistically, MCT2 facilitates _L_-lactate influx into the cortex with or without WS, promoting lactate uptake by neurons and astrocytes. This, in turn, induces MCT2 expression in neurons and activates hypoxia-inducible factor 1α (HIF1α) and VEGFa expression in astrocytes. Together, these findings uncover a previously unrecognized role for neuronal MCT2 in regulating lactate flux, signaling, and vascular remodeling, thereby linking neural activity to metabolic adaptation and vascular development in the neonatal mouse neocortex.

## Introduction

The brain has a high energy demand and depends on a complex cerebral vasculature to meet its energy needs [1]. Alterations in brain vasculature are associated with various disorders, including neurodegenerative diseases [2]. Like blood vessels (BVs) in other tissues, cerebral BVs are composed of endothelial cells (ECs) and supporting cells, such as smooth muscle cells for arteries/arterioles and pericytes for micro-vessels [3]. In contrast to peripheral BVs, the ECs of cerebral BVs directly associate with astrocytes, a most abundant subtype of glial cells in the brain, through their end-feet. Astrocytes thus play essential roles in regulating cerebral BV formation, homeostasis, blood flow, and the integrity of blood-brain barrier (BBB) via their direct contacts and secreted factors [4–6]. Beyond astrocytes, mounting evidences has identified neural activity as a critical regulator in promoting cerebral BV formation and blood flow in a phenomenon known as neurovascular coupling (NVC) [4, 7–9]. For example, acute whisker stimulation (WS) in adult mice increases cortical blood flow [10], while chronic WS in neonatal mice promotes BV formation in the barrel cortex [11]. Conversely, sensory deprivation, such as whisker plucking, reduces BV networks [11, 12]. However, the precise mechanisms by which neural activity induces BV formation remain elusive.

It is important to note that neurovascular coupling (NVC) is closely linked with neurometabolic coupling (NMC), a process that connects changes in neuronal activity with the supply of energy substrates [13, 14]. A key aspect of NMC is the relationship between neural activity and primary energy substrates, such as glucose and lactate. It has been shown that neural activation leads to a rapid decrease in brain glucose levels, accompanied by a simultaneous increase in lactate concentrations [15, 16]. This raises important questions: how does neural activity simultaneously drive a reduction in glucose and an increase in lactate in the brain? How does neural activity coordinate the processes of NMC with BV formation and/or NVC?

Lactate, produced from pyruvate by lactate dehydrogenase (LDH), has long been viewed as a byproduct of aerobic glycolysis. Notable, the LDH reaction operates near thermodynamic equilibrium, with lactate flux primarily regulated by its relative concentration [17, 18]. In the adult brain, lactate is thought to be predominantly generated in astrocytes through glycolysis and acts as a crucial fuel for neurons via the astrocyte-to-neuron lactate (ANLS) shuttle pathway [19–23]. Additionally, lactate functions as a metabolic signaling molecule, influencing neuronal activity and mitochondrial ATP production [24, 25]. During embryonic mouse neocortex development, lactate, likely originating from radial glial progenitors, contributes to the coordination of neurogenesis and vasculature formation [26, 27]. Despite lactate’s abundance in the brain and its induction by neural activity [28, 29], the specific sources, functions, and mechanisms of activity-induced lactate remain poorly understood.

Lactate flux across cell membranes is mediated by monocarboxylate transporters (MCTs), which are members of the solute carrier 16 A (SLC16A) family. These proton-linked membrane proteins transport monocarboxylates such as lactate, pyruvate, and ketone bodies [30, 31]. The SLC16A/MCT family includes MCT1/SLC16A1, MCT2/SLC16A7, MCT3/SLC16A8, and MCT4/SLC16A3, each exhibiting cell- and tissue-specific expression patterns: MCT1 in capillary and venous ECs, MCT2 in neurons, and MCT3 in the retinal pigment epithelium and choroid plexus [32–34]. Basigin (Bsg) and embigin (Emb), transmembrane glycoproteins, facilitate the targeting of MCTs to the plasma membrane [35, 36]. MCT1 and MCT2 are involved in lactate transport in the hippocampus and are essential for learning and memory in rodents [37, 38]. However, their roles, particularly through neuronal MCT2, in regulating lactate signaling, angiogenesis, and NMC remain largely unclear and require further investigation.

Here, we subjected neonatal mice to a chronic WS paradigm, which is known to promote vasculature in the somatosensory (S1) cortex [11, 12, 39]. RNA-seq analysis of the cortex revealed increased expression of MCT1-3, Bsg, and Emb due to WS. We focused on neuronal MCT2 in WS-induced vascular development, and obtained the following findings: (1) increased MCT2 expression in pyramidal neurons of the barrel cortex after WS and its decrease following whisker plucking; (2) essential roles of neuronal MCT2 in enhancing astrocytic VEGFa expression and BV formation in the barrel cortex; (3) WS-induced lactate flux to the cortex being dependent on MCT2; (4) _L_-lactate influx via MCT2 inducing its own expression in neurons; (5) _L_-lactate influx via MCT1 stabilizing HIF1α and inducing VEGFa in astrocytes, likely by inhibiting α-ketoglutarate (α-KG) activation of prolyl hydroxylases (PHs); and (6) subcutaneous _L_-lactate injections mimicking the effect of WS, alleviating the BV deficit in MCT2-deficient mice. Together, these findings underscore MCT2’s pivotal role in both baseline and activity-induced lactate flux and angiogenesis. They provide evidence that _L_-lactate acts as a signaling molecule, enhancing MCT2 expression in neurons, stabilizing HIF1α, and increasing VEGFa in astrocytes, thereby promoting angiogenesis in the neonatal mouse cortex. These results also offer new insights into the association and coordination of neural activity-induced metabolic supply and angiogenesis.

## Results

### Neuronal MCT2 expression linked to neural activity and BV network formation in the developing mouse barrel cortex

To explore mechanisms underlying neural activity-induced BV formation, mice at P14 underwent daily WS for 8 days (Supplementary Fig. 1a). At P21, sections of their S1 cortex were immunostained using antibodies against various BV markers, including EC markers (e.g., CD31, PODXL, IB4), pericyte marker PDGFRβ, and arteriole marker SMA (smooth muscle actin) [40–43]. BV densities labeled by these markers, except SMA, were selectively increased in layers IV-V of the contralateral barrel cortex stimulated by WS, compared to the non-stimulated ipsilateral side (NS) (Supplementary Fig. 1b-j). This increase primarily occurred in BVs with diameters of 5–10 µm (Supplementary Fig. 1d). These results, consistent with previous findings [9, 11, 44], suggest that WS promotes the formation and maturation of BV networks, predominantly capillaries and venules.

After establishing the model, we conducted bulk RNA-seq analysis and identified 363 up- and 138 down-regulated DEGs (differentially expressed genes) in the stimulated S1 cortex (Supplementary Fig. 2a, b). GESA (Gene Enrichment Score Analysis) and GO analysis highlighted top pathways including BV development, angiogenesis, and lactate transporter/metabolism (Supplementary Fig. 2c–f). Specifically, MCT1-3, along with Bsg and Emb, which promote MCT targeting to the plasma membrane [35, 36], were significantly increased by WS (Supplementary Fig. 2b-f). These findings were validated by RT-qPCR (Supplementary Fig. 2g) and Western blot analyses (Supplementary Fig. 2h, i).

**Fig. 1:**
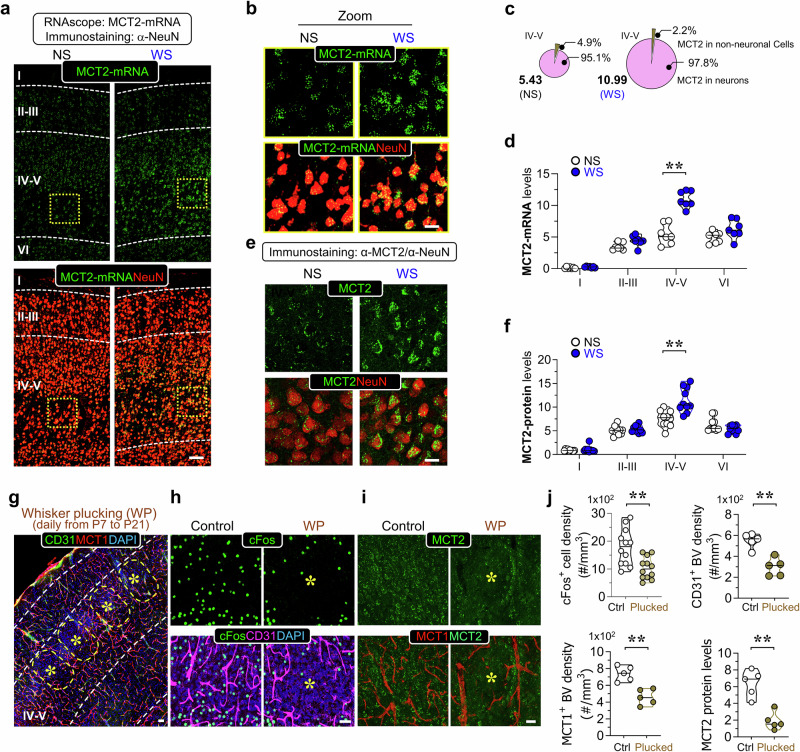
Neuronal MCT2 expression in mouse S1 cortex in response to WS or plucking. **a–d** RNAscope analysis of MCT2-mRNAs, which were co-immunostained with indicated antibodies, in the S1 cortex of WT mice with WS or NS. Representative images **a** and zoom images **b** from layers IV-V (in yellow square) were shown. Quantifications of neuronal MCT2 mRNA distribution [in neurons (MCT2^+^NeuN^+^) and other cells (Mct2^+^NeuN^-^) over total, %] and MCT2-mRNA intensity in layers I-VI were presented in **c** and **d**, respectively. The data in **d** were mean ± SD (*n* = 7 mice per group, **P < 0.01, two-way ANOVA and Bonferroni post comparisons test). **e, f** Co-immunostaining analysis using indicated antibodies in S1 cortex of WT mice with WS or NS. Representative images in layers IV-V **e**, and quantifications of MCT2^+^ intensity per indicated area (mean ± SD, *n* = 10 mice per group, **P < 0.01, two-way ANOVA and Bonferroni’s multiple comparisons test) **f** were shown. **g–j** Representative images of co-immunostaining analysis in the relevant S1 cortex barrel column region with whisker plucking **g**. Representative images of co-immunostaining in the relevant S1 cortex barrel column region with or without whisker plucking **h, i**, and quantifications of cFos^+^ cell density, CD31^+^-BV density, MCT1^+^-BV density, and MCT2 protein levels in whisker plucked and control columns (layers IV-V) (mean ± SD, *n* = 5 ~ 12 from 3 mice per group, **P < 0.01, student’s t-test) **j** were shown. Scale bars = 50 μm.

We then investigated the layer-specific induction of MCT1-3 and the cell types involved in the S1 cortex following WS using RNAscope combined with co-immunostaining analysis. We observed that MCT1-3 were all induced in layers IV-V of the S1 cortex (Fig. 1a–f and data not shown), with varying expression across different cell types (Supplementary Fig. 3). Specifically, MCT1 mRNA was prominently expressed in CD31^+^ ECs (~58%), and to a lesser extent in GLAST1^+^ astrocytes (~16%) and other cell types (~26%) (Supplementary Fig. 3a, b), consistent with previous reports of MCT1 expression primarily in ECs of capillaries and venules [45]. Importantly, the increase in MCT1 mRNA induced by WS was selective to CD31^+^ ECs, with no significant change observed in GLAST1^+^ astrocytes or other cell types (Supplementary Fig. 3c). The elevated MCT1 mRNA levels correlated well with increased ECs following WS, maintaining comparable levels per CD31^+^ EC between NS and WS conditions (Supplementary Fig. 3d).

Unlike MCT1, MCT3 mRNA was detected in various cell types, including GLAST^+^ astrocytes (~15%), NeuN^+^ neurons (~14%), Oligo2^+^ glial cells (~10%), IBA1^+^ microglia (~10%), and cells of unknown identity (~50%) (Supplementary Fig. 3e, f). The increase in MCT3 mRNA induced by WS was most prominent in GLAST1^+^ astrocytes and cells of unknown identity in layers IV-V (Supplementary Fig. 3g).

Distinct from MCT1/3, MCT2 mRNA was specifically expressed in NeuN^+^ neurons (>95%) (Fig. 1a–d). This was confirmed by co-immunostaining analysis (Fig. 1e, f). Further analysis revealed that the majority (60% to 80%) of MCT2^+^ neurons were NeuroD6-Cre^+^ glutamatergic pyramidal neurons (Supplementary Fig. 4a, b). These neurons are a key neuronal subset in the barrel cortex responsive to WS, as indicated by the prominent induction of cFos, an immediate early gene (IEG) and neural activity marker, specifically in NeuroD6-Cre^+^ pyramidal neurons but not in PV (parvalbumin) interneurons in layers IV-V of the S1 cortex following WS (Supplementary Fig. 4c–h). Additionally, the increased MCT2 expression co-localized with induced cFos expression (Supplementary Fig. 4i, j), suggesting their closely associated regulation. These findings underscore the interconnected roles of neural activity, IEG/c-Fos expression, MCT2 induction, and MCT1+ micro vessel density in the S1 cortex.

To further investigate their association, we employed the whisker plucking (WP) model (Fig. 1g). Consistent with previous findings [11], individual whisker plucking led to reductions in neural activity (cFos^+^) and BV density (CD31^+^ and MCT1^+^) in the corresponding barrel columns (Fig. 1h–j). Concurrently, there was a decrease in neuronal MCT2 expression in the whisker-plucked barrel column (Fig. 1h–j). These results provide additional evidence linking neuronal MCT2 expression with neural activity, IEG/cFos expression, and BV density, highlighting the role of MCT2 in neural activity-induced BV network formation.

### Simultaneous induction of neuronal IEG/cFos and MCT2 by MCT2-mediated lactate influx

**Fig. 2:**
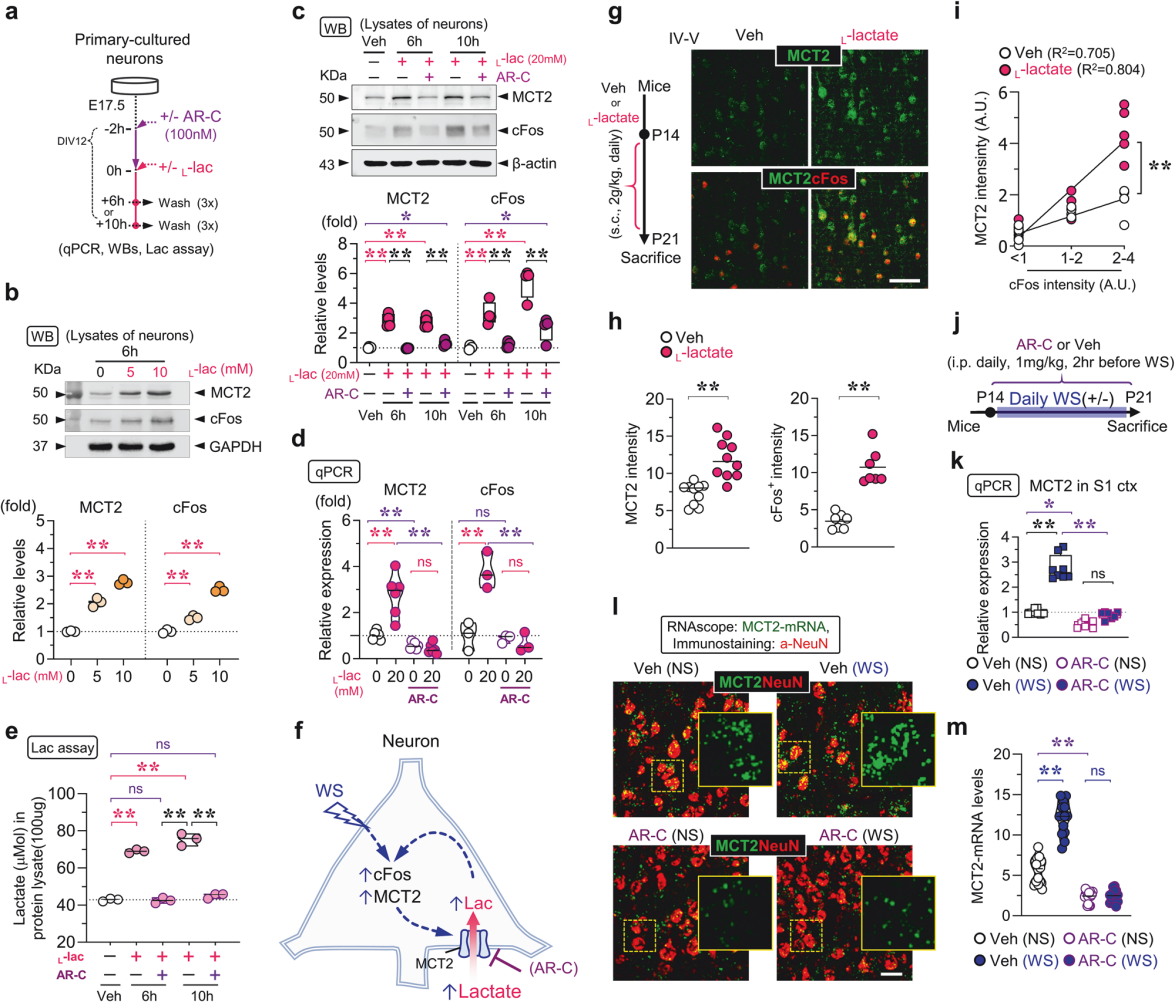
Inductions of neuronal cFos and MCT2 expression in culture and in vivo by MCT2-mediated lactate-influx. **a–e** Exogenous _L_-lactate induced IEG/cFos and MCT2 expressions and _L_-lactate uptake in cultured neurons, which were all abolished by AR-C155858 (AR-C), an inhibitor of MCT2. The schematic of experimental protocol **a**, the representative Western blots and their the quantification of data **b, c**, the qPCR analysis of MCT2 and cFos **d**, and the up-taken _L_-lactate levels **e** were shown. Data in **b** and **d, e** were mea*n* ± SD (*n* = 3–6 mice per group, *P < 0.05, **P < 0.01, one-way ANOVA with Tukey’s Honestly Significant Difference (HSD) test). **f** The schematic of a working model for _L_-lactate or WS induced MCT2 expression in neurons. The blue dotted arrows indicate signaling pathways, and the pink arrow mark the lactate influx. **g, h** Induced neuronal IEG/cFos and MCT2 in S1cortex (layers IV-V) by subcutaneous *(*s.c.) injections of _L_-lactate. The schematic of experimental protocol (**g**, left), the representative images of co-immunostaining analysis of MCT2 (green) with cFos (red) in the S1 cortex (layer IV-V) with vehicle or _L_-lactate injections (**g**, right), the quantifications of MCT2 intensity and cFos^+^ cell intensity **h** were shown. The data in **h** were mea*n* ± SD [*n* = 7 ~ 10 (area in 1x10^2^mm^3^)] from 3 mice per group (**P < 0.01, student’s t-test). **i** Correlation of MCT2 and cFos intensity with fitted linear regression analysis (mea*n* ± SD, *n* = 3 mice per group, R^2^ = Correlation coefficient, **P < 0.01; linear regression difference). **j** The schematic of experimental protocol. **k–m** The qPCR analysis of MCT2 **k** and representative images of RNAscope analysis of MCT2-mRNAs, which were co-immunostained with NeuN antibody **l**, and the quantification [mea*n* ± SD, *n* = 14 from 3 mice per group, *P* < 0.01, one-way ANOVA with Tukey’s HSD test] **m** were shown. Scale bars = 50 μm. The blue star (*) indicates a comparison between with WS or NS. The magenta star (*) indicates a comparison between AR-C inhibitor and vehicle control. The magenta star (*) are comparisons between presence and absence of L-lactate, while the black star (*) indicates a comparison between each indicated experiment group, respectively.

Considering the reports of _L_-lactate regulation of neural activity [24, 46, 47], we investigated the impact of _L_-lactate on MCT2 expression in cultured neurons and in mouse S1 cortex. Primary cortical neurons from mouse embryos were treated with _L_-lactate at various concentrations (e.g., 5-, 10-, and 20 mM) for 6 or 10 h (Fig. 2a). Western blot analysis revealed that _L_-lactate increased levels of cFos and MCT2 in a dose dependent manner (Fig. 2b). Interestingly, the increases in c-Fos and MCT2 detected by both Western blot and RT-PCR analyses were abolished by AR-C155858 (Fig. 2c, d), an MCT1/2 transporter inhibitor [48–50], which blocked the lactate influx into the neurons (Fig. 2e). Given the predominant expression of MCT2 over MCT1 in neurons, these findings suggest that MCT2-mediated influx of _L_-lactate is crucial for inducing c-Fos and MCT2 expression in neurons, implying a positive feed-forward mechanism(Fig. 2f).

Next, we investigated whether increasing circulating _L_-lactate levels in vivo could induce MCT2 expression in neurons of the S1 cortex. Neonatal mice were subcutaneously (s.c.) injected with _L_-lactate (2 g/kg bodyweight) once daily for 8 days (Fig. 2g, left). An elevated neuronal MCT2 levels in the S1 cortex was detected following _L_-lactate injections, compared to vehicle controls, correlating closely with the induction of cFos by co-immunostaining analysis (Fig. 2g–i). These findings provide compelling in vivo evidence that _L_-lactate stimulates the expression of cFos and MCT2 in neurons in vivo.

We further explored whether the induction of neuronal MCT2 by WS relies on MCT1/2-mediated lactate flux. Mice were intraperitoneally injected with AR-C155858 and subsequently exposed to WS (Fig. 2j). In vehicle-injected mice, RT-PCR and RNAscope analysis detected increased MCT2 mRNA levels in the cortex following WS (Fig. 2k–m). However, AR-C15858 not only decreased baseline MCT2 mRNA levels in the S1 cortex but also prevented WS-induced MCT2 expression (Fig. 2k–m). These findings highlight the necessity of MCT1/2 transporter activity for both basal and WS-induced MCT2 expression. Together, these results suggest that MCT2-mediated lactate influx into neurons serves as a signaling mechanism that reinforces neural activity-induced MCT2 expression, establishing a positive feed-forward loop (Fig. 2f).

### Enhanced BV network formation by MCT2 in baseline and activity-induced conditions

We proceeded to investigate the role of MCT2 in neural activity-induced angiogenesis. Initially, we assessed the impact of AR-C155858 on the BV increase induced by WS. As depicted in Supplementary Fig. 5a, b, the elevation in MCT1^+^ BVs due to WS was significantly diminished in mice injected with AR-C155858 compared to vehicle-injected mice, indicating a requirement for MCT1/2 activity in this induction.

Considering that AR-C155858 was administered intraperitoneally to the mice, potentially inhibiting both MCT1 and MCT2 transporters systemically, we investigated its effects on body weight and lactate levels in serum, peripheral tissues (such as liver and TA muscles), and the S1 cortex. Surprisingly, the AR-C155858-treated mice showed little to no change in body weight (Supplementary Fig. 5c), in TA muscle and liver masses (Supplementary Fig. 5d), or in _L_-lactate levels in serum and liver (Supplementary Fig. 5e–h). However, the treatment notably reduced lactate levels in the S1 cortex and TA muscles (Supplementary Fig. 5g, h). These findings suggest that MCT1 and MCT2 in the S1 cortex and TA muscles may be more susceptible to AR-C155858 treatment. To explore this further, we performed Western blot analysis of MCT1, and MCT2 proteins in homogenates of S1 cortex, TA muscles, and liver. Interestingly, AR-C155858 treatment led to reductions in MCT1 and MCT2 levels in the S1 cortex, but not in the liver (Supplementary Fig. 5i, j). Consistent with the reduced lactate levels in the TA muscles, a decrease in MCT2 in the TA muscles, but not MCT1 was detected (Supplementary Fig. 5i, j). These results support the hypothesis that AR-C155858 preferentially affects MCT2 and MCT1 in the cortex and MCT2 in TA muscles.

Given AR-C155858’s inhibitory effects on both MCT1 and MCT2, to assess the specific function of neuronal MCT2, it is important to generate a specific MCT2 suppressor. To this end, we generated a lentivirus expressing shRNA targeting MCT2. This approach effectively reduced MCT2 expression, as confirmed by both Western blot and immunostaining analyses (Supplementary Fig. 6a–d). These results also demonstrated the specificity of the MCT2 antibody. The antibody recognized a single band at the expected molecular weight in Western blot analysis, which was diminished following shRNA-MCT2 virus infections (Supplementary Fig. 6a, b) and increased upon overexpression of the MCT2-mCherry fusion protein (Supplementary Fig. 6e–g). Additionally, co-immunofluorescence analysis revealed overlapping signals between the MCT2 antibody and the mCherry from the overexpressed MCT2-mCherry fusion protein (Supplementary Fig. 6h, i), further supporting the antibody’s specificity.

**Fig. 3:**
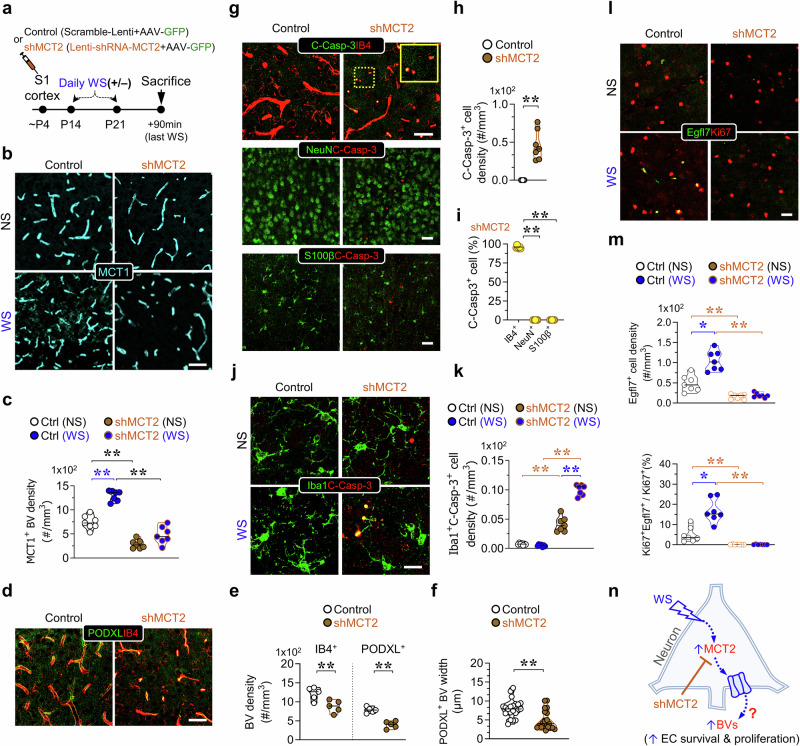
Requirement of MCT2 for basal and WS-induced BV formation and EC survival. **a** The schematic of the experimental protocol. Lentivirus of MCT2-shRNA (MCT2-shRNA) or scrambled shRNA particles, mixed with AAV-hSyn-GFP virus (to mark injected brain region), were injected into the S1 cortex of WT mice. **b, c** Representative immunostaining images of MCT1^+^ (cyan) BVs in the layers IV-V of viruses infected regions in the of S1 cortex in **b**, and quantification of MCT1^+^-BV densities in layers IV-V (*n* = 7 from 3 mice per group, mean ± SD, **P < 0.01, one-way ANOVA with Tukey’s HSD test) in **c** were shown. **d–f** Representative images of co-immunostaining analysis of Podocalyxin (PODXL) and Isolectin GS-IB4 (red) BVs in layers IV-V of the S1 cortex in **d**, and quantifications of PODXL^+^ and IB4^+^ BV densities in **e**, and PODXL^+^ BV diameters in **f** (mean ± SD, *n* = 7 from 3 mice per group, **P < 0.01, student’s t-test) were shown. **g–i** Representative images of co-immunostaining with indicated antibodies in **g**, and the quantifications (mean ± SD, *n* = 7 from 3 mice per group, **P < 0.01, student’s t-test, or one-way ANOVA with Tukey’s HSD test) in **h, i** were shown. **j, k** Representative images of the co-immunostaining analysis using indicated antibodies in (**j**), and the quantifications of the phagocytic microglia (Iba1^+^ C-Casp-3^+^) densities (mean ± SD, *n* = 7 from 3 mice per group, **P < 0.01, one-way ANOVA with Tukey’s HSD test) in **k** were shown. **l, m** Representative images of co-immunostaining analysis using indicated antibodies in **l**, and the quantifications of Egfl7^+^ cell density and ki67^+^Egfl7^+^ over total ki67^+^ cells (%)(mean ± SD, n = 7 from 3 mice per group, *P < 0.05, **P < 0.01, one-way ANOVA with Tukey’s HSD test) in **m** were shown. **n** A schematic of a working model. The blue dotted arrows indicate signaling pathways. Scale bars = 50 μm. The blue star (*) indicates a comparison between with WS or NS. The brown star (*) indicates a comparison between shRNA-MCT2 or scramble shRNA control, while the black star (*) indicates a comparison between each indicated experiment group, respectively.

We then injected control (scramble) and shRNA-MCT2 viruses into the S1 cortex of P4 pups and examined their effect on BV increase induced by WS (Fig. 3a). Indeed, the increase in MCT1^+^ BV density triggered by WS was abolished by shRNA-MCT2 (Fig. 3b, c), confirming the essential role of MCT2 in this process. Notably, shRNA-MCT2 reduced MCT1^+^ BV density in the absence of WS (Fig. 3b, c), suggesting that MCT2 is involved in the basal development and/or maintenance of BVs.

Furthermore, we confirmed the role of neuronal MCT2 in BV formation and/or maintenance by co-immunostaining analysis with other EC markers, such as PODXL and IB4. Using shRNA-MCT2, we observed reductions in both PODXL^+^ and IB4^+^ BV densities (Fig. 3d, e). Remarkably, PODXL^+^ BVs in shRNA-MCT2 injected samples exhibited narrower widths compared to scramble controls, resembling “string vessels” (Fig. 3d, f), a phenotype associated with compromised EC survival. To investigate this further, we examined the presence of active or cleaved caspase 3 (C-Casp-3) in IB4^+^ ECs. In shRNA-MCT2-injected barrel cortex, C-Casp-3 was detectable in IB4^+^ ECs but absent in NeuN^+^ neurons, S100β^+^ glial cells, or IBA1^+^ microglia (Fig. 3g–k). Interestingly, upon WS, C-Casp-3 was associated with IBA1^+^ microglial processes (Fig. 3j, k). Additionally, shRNA-MCT2 abolished the induction of tip EC proliferation (Egfl7^+^Ki67^+^) observed during WS (Fig. 3l, m). These findings suggest that compromised EC survival and impaired tip EC proliferation contribute to the reduced BV density in shRNA-MCT2-treated mice. Overall, these results support the role of neuronal MCT2 in promoting EC proliferation and survival (Fig. 3n).

**Fig. 4:**
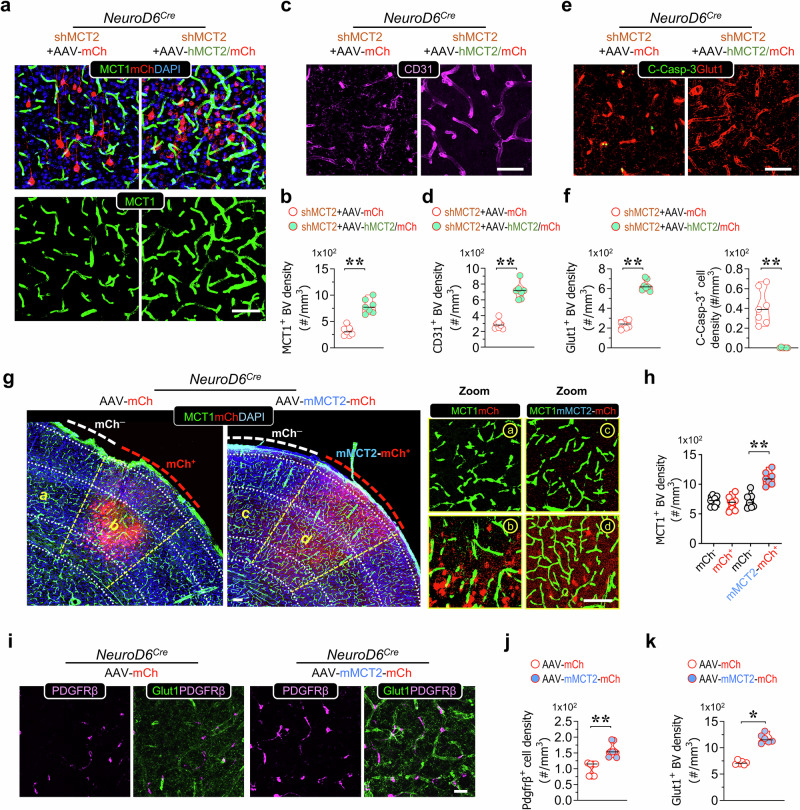
Expressing neuronal MCT2 mimicking WS and increasing BV network formation. **a, b** Representative images of co-immunostaining analysis of MCT1 with mCherry in human-MCT2 virus infected S1 cortex in NeuroD6-Cre mice **a** and quantification of MCT1^+^-BV density **b** were shown (mean ± SD, *n* = 7 from 3 mice per group, **P < 0.01, student’s *t*-test). **c–f** Representative images of immunostaining analysis with indicated antibodies in **c,** **e**, and quantifications (mean ± SD, *n* = 7 from 3 mice per group, **P < 0.01, student’s *t*-test) in **d, f** were shown. **g, h** Representative images of co-immunostaining analysis of mouse-MCT2 and mCherry, with DAPI in mMCT2-mCh fusion virus infected S1 cortex in NeuroD6^Cre^ mice **g**, and quantification of MCT1^+^-BV density **h** (mean ± SD, *n* = 7 from 3 mice per group, **P < 0.01, one-way ANOVA with Tukey’s HSD test) were shown. **i–k** Representative images of immunostaining analysis of PDGFRβ (magenta) and Glut1^+^(green)-BV in **i**, and quantifications of PDGFRβ^+^ pericyte density **j** and Glut1^+^-BV density **k** (mean ± SD, *n* = 7 from 3 mice per group, *P < 0.05, **P < 0.01, student’s *t*-test) was shown. Scale bars = 75 μm.

To further validate that the deficits in BVs resulted specifically from neuronal MCT2 suppression by shRNA-MCT2, and not from off-target effects, we generated AAV-hSyn-DIO-hMCT2-T2A-mCherry. This virus expresses human MCT2 (hMCT2) in a Cre-dependent and shRNA-MCT2-resistant manner (Supplementary Fig. 7). In NeuroD6-Cre mice, we co-injected shRNA-MCT2 lentivirus with AAV-hSyn-DIO-hMCT2-T2A-mCherry or a control virus (AAV-hSyn-DIO-mCherry) into the S1 cortex, and assessed their effects on BVs (Fig. 4a–f). The BV densities marked by MCT1, CD31, or Glut1 were elevated in regions where shRNA-MCT2 was co-expressed with hMCT2, compared to those co-infected with shRNA-MCT2 and the control virus (Fig. 4a–f). Additionally, the EC death marker (C-Casp-3^+^IB4^+^) was reduced in areas where hMCT2 was co-expressed with shRNA-MCT2 (Fig. 4e, f). Western blot analysis confirmed an increase in MCT1 levels in response to hMCT2 expression (Supplementary Fig. 7c, d). These findings provide strong evidence that the deficits in BVs observed with shRNA-MCT2 were specifically due to the loss of neuronal MCT2, rather than off-target effects of shRNA-MCT2, underscoring critical roles of neuronal MCT2 in maintaining EC survival and BV density in the developing S1 cortex.

We next investigated whether increasing MCT2 levels in neurons could replicate the effects of WS on BVs. The S1 cortex of NeuroD6-Cre mice was injected with AAV-hSyn-DIO-mMCT2-mCherry, which expresses a mouse MCT2-mCherry fusion protein in a Cre-dependent manner (Supplementary Fig. 6e–i). As shown in Fig. 4g–k, the density of MCT1^+^ BVs was notably increased in regions where mMCT2-mCherry was expressed, compared to adjacent regions within the same mouse that were not infected with AAV-hSyn-DIO-mMCT2-mCherry, as well as compared to different mouse cortices infected with a control virus. Consistent with this finding, Western blot analysis showed an elevation in MCT1 protein levels upon neuronal expression of mMCT2-mCherry (Supplementary Fig. 6f, g). These results indicate that augmenting MCT2 levels in neurons is sufficient to enhance cerebral BV density, thereby mimicking the effects observed with WS.

### Facilitating baseline- and activity-induced astrocytic VEGFa expression by MCT2

**Fig. 5:**
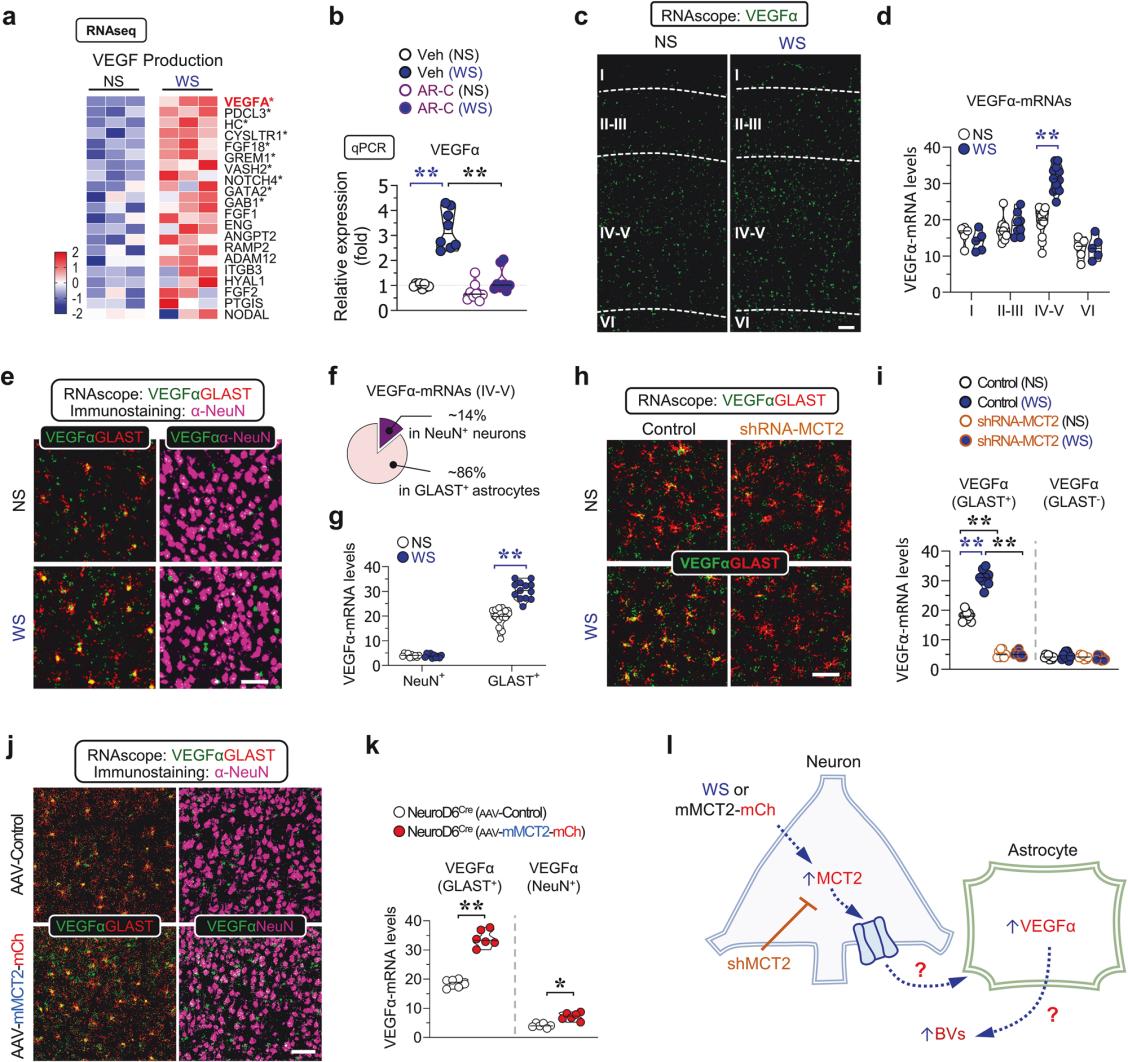
Neuronal MCT2 for basal and activity induced astrocytic VEGFa. **a** Heatmap analysis of RNA-seq showing VEGFa production pathway gene expression profiles (DEGs, differential expression genes) in the S1 cortex of WT mice with WS or NS. All the data were Z-transformed. Red means increased, and blue represents decreased expression. *P < 0.05 (*n* = 3 mice). **b** qPCR analysis of VEGFa-mRNA levels in the S1 cortex of the mice injected with or without MCT1/2 inhibitor (AR-C155858) and in the presence of WS or NS. Data were normalized by each group’s GAPDH ct-value and shown as relative expression (fold) (mean ± SD, *n* = 8 from 3 mice per group **P < 0.01, one-way ANOVA with Tukey’s HSD test). **c, d** RNAscope analysis showing VEGFa-mRNA distribution and levels in the S1 cortex (layer I-VI) with WS or NS. Representative images **c** and quantifications of VEGFa-mRNA levels **d** (mean ± SD, n = 5 ~ 12 from 3 mice per group, **P < 0.01, two-way ANOVA and Bonferroni’s multiple comparisons test) were shown. **e–g** RNAscope analysis showing VEGFa-mRNAs in GLAST (red) or NeuN (magenta) positive cells in S1 cortex with WS or NS. Representative images **e**, quantifications in VEGFa-mRNA distribution **f** and levels **g** in NeuN^+^ neurons and GLAST^+^ astrocytes with WS or NS (mean ± SD, *n* = 13 from 3 mice per group, **P < 0.01, Student’s t-test) were shown. **h, i** RNAscope analysis of VEGFa-mRNAs in GLAST^+^ (red) astrocytes in S1 cortex with injections of shRNA-MCT2 or scramble viruses and with WS or NS. Representative images **h** and quantification of VEGFa-mRNA levels in GLAST^+^ cells and GLAST^-^ cells **i** (mean ± SD, n = 8 from 3 mice per group, **P < 0.01, one-way ANOVA with Tukey’s HSD test) were shown. **j, k** RNAscope analysis of VEGFa-mRNAs in AAV-MCT2-mCh virus infected or un-infected regions of the S1 cortex. Representative images **j**, and quantifications of VEGFa-mRNA intensity per GLAST^+^ cell or NeuN^+^ Cell in S1 cortex **k** (mean ± SD, *n* = 6 from 3 mice per group, *P < 0.05, **P < 0.01, Student’s t-test) were shown. **i** schematic of the working model. The blue dotted arrows mark the signaling pathways. Scale bars = 50 μm. The blue star (*) denotes a significant difference between WS and NS, while the black star (*) indicates a comparison between each indicated experiment group, respectively.

To explore how neuronal MCT2 increases BV density, we investigated whether VEGFa, a well-known angiogenic growth factor, is influenced by WS and MCT2, and whether it contributes to the induced BV formation. Heat map analysis of our RNA-seq data highlighted genes involved in angiogenesis, showing that VEGFa expression was up-regulated by WS (Fig. 5a). This finding was confirmed by RT-PCR (Fig. 5b) and RNAscope analysis (Fig. 5c, d). Importantly, the increase in VEGFa expression was abolished by AR-C155858 injections, indicating a dependence on MCT1/2 activity (Fig. 5b). Interestingly, most VEGFa mRNAs (~86%) were found in GLAST1^+^ astrocytes, with the remainder (~14%) in NeuN^+^ neurons. Following WS, the increase in VEGFa mRNAs was observed primarily in GLAST1^+^ astrocytes in layers IV-V, rather than in neurons (Fig. 5c–g). Suppression of neuronal MCT2 with its shRNA reduced basal levels of VEGFa mRNAs in astrocytes and prevented WS from enhancing VEGFa expression (Fig. 5h, i). Notably, neuronal VEGFa mRNA levels were unaffected by shRNA-MCT2 (Fig. 5i). These results demonstrate the necessity of neuronal MCT2 for WS-induced expression of astrocytic VEGFa.

We then investigated whether increasing neuronal MCT2 alone is sufficient to induce VEGFa expression in astrocytes. Consistent with BV formation results, enhancing MCT2 levels in neurons (via injection of AAV-hSyn-DIO-mMCT2-mCherry into the S1 cortex of NeuroD6-Cre mice) increased VEGFa mRNA levels in astrocytes compared to control virus-injected regions (Fig. 5j, k). These findings establish the sufficiency of neuronal MCT2 to induce VEGFa expression in astrocytes, implicating astrocytic VEGFa in WS/MCT2-induced BV formation (Fig. 5l).

To assess the functional role of VEGFa in WS/MCT2-induced BV formation, we initially examined whether VEGFa signaling is activated under these conditions. Indeed, phosphorylated VEGFR2 (p-VEGFR2), a key downstream event of VEGFa signaling, was significantly increased by neuronal mMCT2-mCherry expression or WS (Supplementary Fig. 8), and the increased p-VEGFR2 was predominantly associated with BVs (Supplementary Fig. 8c, d).

Next, we investigated whether VEGFa is essential for induced BV formation by generating lentivirus expressing shRNA targeting VEGFa, which reduced VEGFa mRNA levels in naive mice with or without WS (Supplementary Fig. 9a, b). Importantly, shRNA-VEGFa dramatically reduced MCT1^+^ or CD31^+^ BVs (Supplementary Fig. 9c–f), increased EC death (Supplementary Fig. 9g, h), and decreased EC proliferation (Supplementary Fig. 9g, i), similar to the effects observed with shRNA-MCT2 (Fig. 3h, i). These results underscore the critical role of VEGFa in mediating WS/MCT2-induced BV formation, as well as in promoting EC survival and maintenance (Supplementary Fig. 9j).

### _L_-lactate induction of astrocytic VEGFa likely by inhibiting α-KG activation of prolyl hydroxylase and stabilizing HIF1α

Building upon our discoveries that WS or neuronal MCT2 enhances astrocytic VEGFa expression and BV formation in non-cell autonomous manner, and considering reports for lactate as a diffusible signaling molecule involved in angiogenesis [27, 51, 52], we investigated if and how lactate influences astrocytic VEGFa expression.

**Fig. 6:**
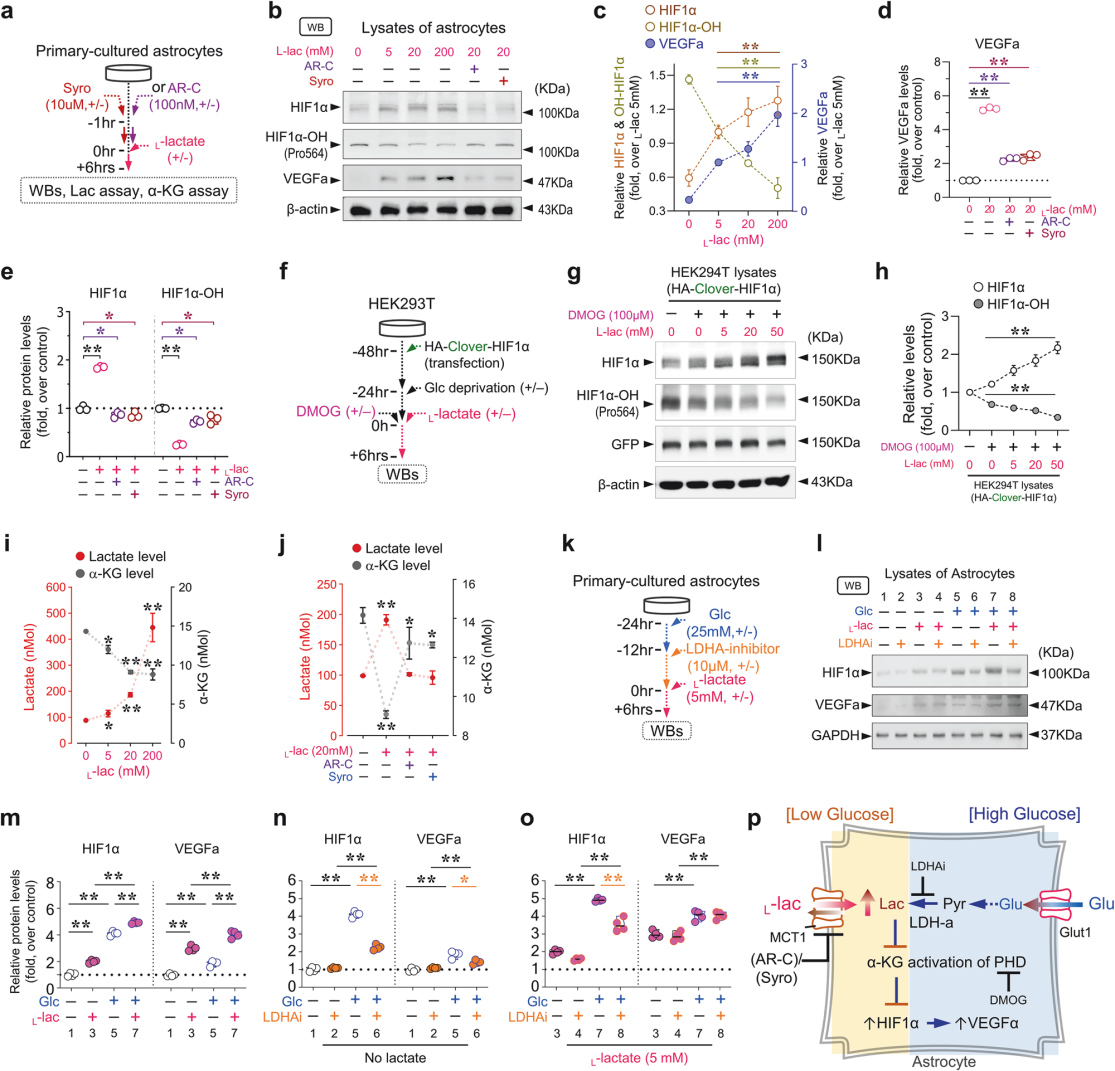
Induction of HIF1α and VEGFa in astrocytes by MCT1 mediated _L_-lactate-influx. **a** A schematic of experimental protocol in primary-cultured astrocytes with indicated treatments. **b** Representative Western blots of cell lysates from cultured astrocytes with indicated conditions and antibodies. **c–e**. Quantifications of data in **c**, **d**, and **e** (mean ± SD, *n* = 4 independent experiments, *P < 0.05, **P < 0.01, one-way ANOVA with Tukey’s HSD test). **f–h** A schematic of experimental protocol in transient transfection of HA-Clover-HIF1α in HEK293T cells with indicated treatment **f**. Representative Western blots of cell lysates with indicated conditions and antibodies **g** and quantifications **h** (mean ± SD, *n* = 3 independent experiments, **P < 0.01, one-way ANOVA with Tukey’s HSD test). **i, j** Quantifications of colorimetric lactate levels **f, g** and α-KG levels **f, g** from culture astrocytes with indicated conditions. The data were mean ± SD (mean ± SD, n = 3 independent experiments, *P < 0.05, **P < 0.01, one-way ANOVA test with Tukey’s HSD test). **k–o** A schematic of experimental protocol in primary-cultured astrocytes with indicated treatments **k**. Representative Western blots of cell lysates from cultured astrocytes with indicated conditions and antibodies **l** and quantifications **m–o** (mean ± SD, *n* = 4 independent experiments, **P < 0.01, student’s t-test). The brown star (*), purple star (*), and magenta star (*) are comparisons between inhibitors, LDHi, AR-C, and Syro, respectively. The black star (*) indicates a comparison between each indicated experiment group. **p** The schematic of a working model for increased intracellular _L_-lactate (by glycolysis pathway or MCT1-mediated lactate influx) to induce HIF1α and VEGFa in astrocytes.

To test this, we first explored the effects on VEGFa and HIF1α an essential transcriptional factor for VEGFa expression [53], in primary cultured astrocytes treated with varying concentrations of exogenous _L_-lactate (Fig. 6a). Western blot analysis revealed dose-dependent increases in HIF1α, and VEGFa by _L_-lactate (Fig. 6b, c), which were abolished by treatment with either the MCT1/2 inhibitor AR-C155858 or the MCT1/4 inhibitor Syrosingopine (Fig. 6b, d, e). These results support the view for MCT1-mediated _L_-lactate influx into astrocytes to induce HIF1α and VEGFa expression.

To explore how _L_-lactate influx affects HIF1α, we examined hydroxylated HIF1α (HO-HIF1α), a critical step in HIF1α ubiquitination and proteasomal degradation [54, 55]. Notably, the increase in HIF1α induced by _L_-lactate was associated with a decrease in HO-HIF1α levels (Fig. 6b, c), suggesting that _L_-lactate may stabilize HIF1α by inhibiting its hydroxylation, thereby promoting VEGFa expression in astrocytes. The stabilization of HIF1α by _L_-lactate appeared to be a general function, not only observed in astrocytes, but also in HEK293 cells (Fig. 6f, h) and tumor cells [56–58].

Considering that PHD (prolyl hydroxylase domain) is the key enzyme responsible for HIF1α hydroxylation, whose activity can be influenced by not only hypoxia, but also α-KG levels and iron availability [59, 60], we measured α-KG levels in astrocytes in response to _L_-lactate treatments. Interestingly, the α-KG levels were decreased dose-dependently with increasing _L_-lactate treatments, showing an inverse correlation with lactate levels (Fig. 6i). Moreover, treatment with AR-C155858 or Syro abolished the decrease in α-KG levels in astrocytes by lactate (Fig. 6j). These results suggest that MCT1-mediated lactate influx may reduce the α-KG activation of PHDs to stabilize HIF1α.

We further investigated the effect of lactate on HO-HIF1α in HEK293 cells in the presence of dimethyloxalylglycine (DMOG), an analogue of α-KG that inhibits PHD (Fig. 6f). As expected, DMOG (0.1 mM) reduced HO-HIF1α (Fig. 6g, h). Unexpectedly, _L_-lactate treatment could further suppress HO-HIF1α and increase HIF1α in a dose-dependent manner (Fig. 6g, h), suggesting a synergistic effect and implicating _L_-lactate functioning at a different site from that of DMOG. Given our findings that lactate influx reduces α-KG levels, we propose that _L_-lactate inhibits PHD and stabilizes HIF1α likely by suppressing α-KG production (Fig. 6p-left).

According to the ANLS hypothesis [19–23], astrocytes produce more lactate through glycolysis than neurons, making them a primary source of lactate for efflux [23, 61, 62]. We thus investigated whether lactate production in astrocytes influences VEGFa and HIF1α levels. Primary astrocytes were cultured with or without glucose (25 mM) and treated with or without the LDHa inhibitor, GSK 2837808 A, to block lactate production from pyruvate (Fig. 6k). Western blot analysis showed that astrocytes cultured with glucose had higher levels of VEGFa and HIF1α compared to glucose-deprived cultures (Fig. 6l, m). Inhibition of LDHa eliminated the glucose-induced increases in VEGFa and HIF1α (Fig. 6l, n), but had little effect on the exogenous lactate-induced HIF1α and VEGFa (Fig. 6o). Together, these findings support the model depicted in Fig. 6p, in which elevated intracellular _L_-lactate levels in astrocytes, resulting from glycolytic production or increased lactate influx, stabilize HIF1α by inhibiting α-KG-mediated activation of PHD. This inhibition prevents HIF1α degradation and promotes VEGFa expression.

### Activity-induced lactate flux to the S1 cortex in a MCT2 dependent manner

Our findings that lactate increases HIF1α and VEGFa through its influx into astrocytes are intriguing, raising several questions. Specifically, are the extracellular lactate levels in the neonatal S1 cortex regulated by WS/neuronal MCT2? Where is the extracellular lactate coming from?

**Fig. 7:**
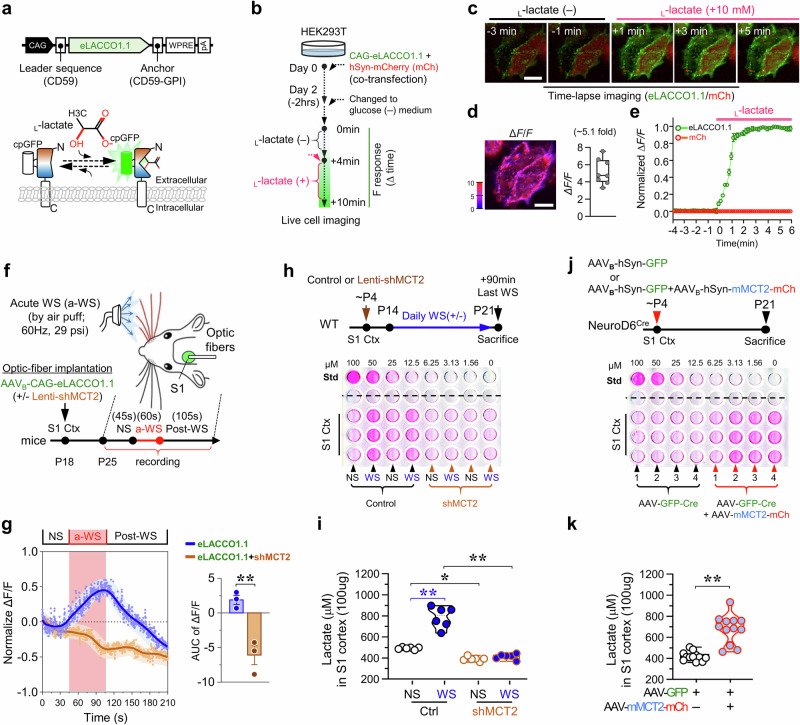
Neuronal MCT2 for basal and activity-induced lactate-flux in the S1 cortex. **a** A schematic representation of the biosensor for extracellular _L_-lactate (eLACCO1.1). **b** An illustration of the in vitro time lapse image analysis in live HEK293T cells expressing eLACCO1.1 biosensor and mCherry. **c–e** The representative images **c**, the quantification of Δ*F*/*F* (*n* = 8 cells) **d**, and the time-course of the fluorescence response of eLACCO1.1 to _L_-lactate stimulation **e** were shown. Scale bars = 20 μm. **f** An illustration of the in vivo optic-fiber photometry analysis of S1 cortex (+/- shRNA-MCT2) with or without acute WS. **g** Representative traces of the fluorescence response (Normalized Δ*F/F*)(left); and the quantification of the traces (right) (AUC = area under the curve) (mean ± SEM, n = 3 mice per group, **P < 0.01, Student’s t-test). **h, i** Colorimetric lactate assay showing increased _L_-lactate levels in the S1 cortex by chronic WS also in a MCT2 dependent manner. The schematic of the experimental protocol **h** and the quantification of data (**i**) were shown. The data were mean ± SD (6 mice per group, * *P* < 0.05, **P < 0.01; one-way ANOVA with Tukey’s HSD test). **j, k** Colorimetric lactate assay showing increased _L_-lactate levels in the S1 cortex by expressing of neuronal MCT2-mCherry fusion protein. The schematic of the experimental protocol **j** and the quantification of data **k** were shown. The data shown were mean ± SD (triplet repeats from 4 mice per group, **P < 0.01; **P < 0.01, student’s t-test). The blue star (*) denotes a significant difference between WS and NS, while the black star (*) indicates a comparison between each indicated experiment group, respectively.

To address whether extracellular lactate levels in the S1 cortex are regulated by WS/neuronal MCT2, we utilized a lactate biosensor (AAV-CAG-eLACCO1.1) [63], where cpGFP fluorescence is activated by extracellular lactate, enabling visualization in live cells and animals (Fig. 7a). Initially, we validated the biosensor’s response to _L_-lactate in HEK293T cells transiently expressing this biosensor along with mCherry (Fig. 7b). Time-lapse imaging showed an increase in cpGFP fluorescence upon _L_-lactate treatment, while mCherry fluorescence remained unchanged (Fig. 7c–e). Subsequently, we used fiber photometry to monitor cpGFP signals in the live mouse S1 cortex, where AAV-CAG-eLACCO1.1 was co-injected with shRNA-MCT2 or control virus (Fig. 7f). Following acute WS, cpGFP signals were induced in the S1 cortex and returned to baseline levels after WS cessation (Fig. 7g), consistent with reports of induced _L_-lactate flux to the S1 cortex upon stimulation [64]. Importantly, WS-induced _L_-lactate flux was abolished in the S1 cortex co-injected with shRNA-MCT2 virus (Fig. 7g), indicating a dependence on MCT2 for this event.

We further validated above findings by measuring _L_-lactate concentrations in the S1 cortex with or without daily WS (for 8 days) using a colorimetric lactate assay. The _L_-lactate levels were significantly elevated in the S1 cortex following WS compared to the unstimulated cortex within the same mouse brain (Fig. 7h, i). Importantly, this increase was abolished by injection of shRNA-MCT2 lentivirus (Fig. 7h, i) or by treatment with the MCT1/2 inhibitor AR-C155858 (Supplementary Fig. 5g, h). These results, in line with recent reports [48, 49], provide additional evidence supporting the role of MCT2 in mediating WS-induced lactate accumulation in the brain.

Similar to the reductions observed in astrocytic VEGFa expression and BV density, both AR-C155858 treatment and shRNA-MCT2 injection decreased lactate levels in the S1 cortex under basal conditions (Fig. 7i and Supplementary Fig. 5g, h). This underscores the necessity of MCT2 for maintaining not only astrocytic VEGFa and BV density but also lactate levels or homeostasis in the S1 cortex under basal conditions.

Furthermore, we observed that lactate levels were increased in the S1 cortex upon expression of mMCT2-mCherry compared to controls (Fig. 7j, k), demonstrating that neuronal MCT2 is sufficient to enhance lactate levels in this brain region. Taken together, these findings suggest that neuronal MCT2 is both necessary and sufficient to enhance extracellular lactate levels in the S1 cortex under both basal and WS conditions.

### Leveraging circulating L-lactate to enhance lactate flux to the S1 cortex and to alleviate angiogenic deficit in MCT2-deficient mice

To address the source of extracellular lactate in the S1 cortex, we measured and compared lactate concentrations in the S1 cortex with those in peripheral organs (e.g., liver and TA muscles), primary cultured neurons and astrocytes, and mouse serum samples (Supplementary Fig. 5g, h and Supplementary Fig. 10). Lactate levels in the S1 cortex (~500 μM/100 μg lysates) were lower than those in muscle (~560 μM/100 μg lysates) or liver (~2300 μM/100 μg lysates), but higher than in cultured astrocytes (~100 μM/100 μg lysates) or neurons (~43 μM/100 μg lysates) (Supplementary Fig. 10e).

Additionally, serum lactate concentration was higher in neonatal (P21) mice (~2.7 mM/L) compared to young adult (2-MO) mice (~1.8 mM/L), while serum glucose levels were lower in neonates than in adults (Supplementary Fig. 10c–e). Together, these findings suggest that blood-borne lactate could be a significant source for the developing S1 cortex.

**Fig. 8:**
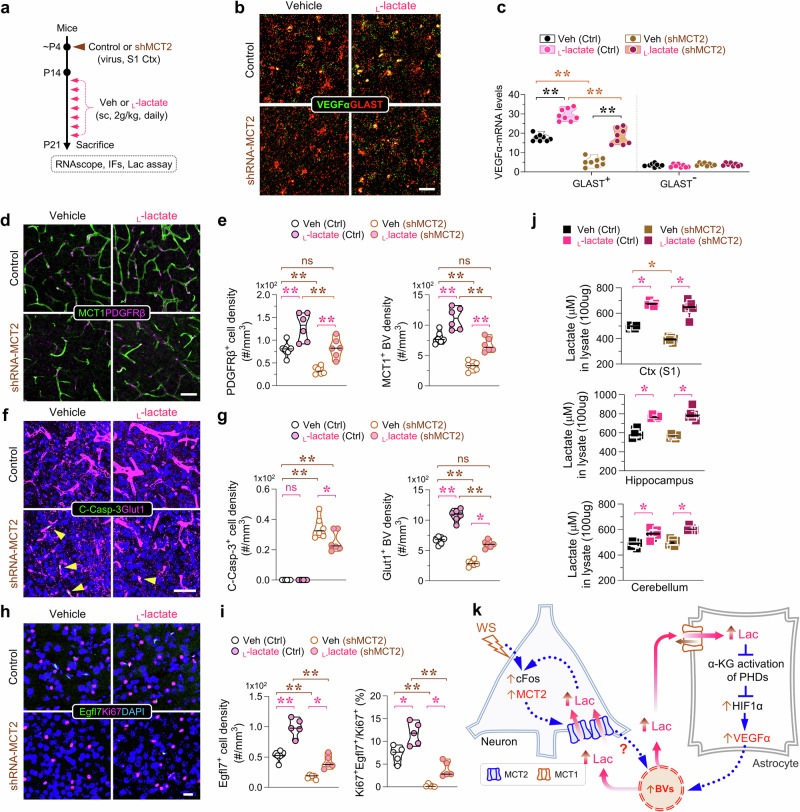
Induction of BV density in S1 cortex by increasing circulating _L_-lactate in a MCT2 independent manner. **a–c** Inducing astrocytic VEGFa expression in S1 cortex by subcutaneous (s.c.) injection with _L_-lactate. A schematic of the experimental protocol. Lentivirus of MCT2-shRNA or scrambled shRNA particle was injected into the S1 cortex at P4, and mice were subcutaneously injected with vehicle or _L_-lactate (2 g/kg, daily, for 8 days) **a**. Representative images of co-RNAscope analysis of VEGFα-mRNAs (green) with GLAST-mRNA (red) **b**. Quantifications of data in **c** (mean ± SD, 9 mice per group, *P < 0.05, **P < 0.01, one-way ANOVA with Tukey’s HSD test). **d,** **f,** **h** Representative images of co-immunostaining analysis with indicated antibodies. DAPI was used as the counterstain. **e,** **g,** **i** Quantifications of the data in a, c, and e, respectively. Mean ± SD, *n* = 6 from 3 mice per group, *P < 0.05, **P < 0.01, one-way ANOVA with Tukey’s HSD test. **j** Quantifications of colorimetric lactate measurements in S1 cortex, hippocampus, and cerebellum lysates, respectively (mean ± SD, 6 mice per group, *P < 0.05, **P < 0.01, one-way ANOVA with Tukey’s HSD test). **k** The schematic of a working model for circulating _L_-lactate to mitigate BV deficits induced by MCT2 deficiency. The blue dotted arrows mark the signaling pathways, and pink and brown arrows mark the lactate influx and efflux, respectively. Scale bars = 50 μm. The magenta star (*) indicates a comparison between with veh or L-lactate treatment and brown star (*) indicates a comparison between control and shMCT2, respectively.

To further test above view, we explored whether increasing circulating _L_-lactate could mimic WS/neuronal MCT2 to stimulate astrocytic VEGFa expression and angiogenesis in vivo. Neonatal mice received subcutaneous injections of _L_-lactate (2 g/kg body weight; once daily for 8 days) to elevate serum lactate levels (Fig. 8a). RNAscope analysis demonstrated heightened VEGFa-mRNA expression predominantly in GLAST1^+^ astrocytes in the brain/S1 cortex following _L_-lactate injections compared to controls (Fig. 8b, c). Interestingly, in the S1 cortex injected with shRNA-MCT2 virus, _L_-lactate injections still increased astrocytic VEGFa expression (Fig. 8b, c), contrasting with the MCT2-dependent induction observed with WS (Fig. 5h, i). These findings provide in vivo evidence that the circulating _L_-lactate induces astrocytic VEGFa expression in an MCT2-independent manner.

Given the increase in astrocytic VEGFa through subcutaneous injections of _L_-lactate in an MCT2-independent manner, we investigated whether elevating circulating _L_-lactate could mitigate BV deficits induced by MCT2 deficiency. Indeed, along with the rise in astrocytic VEGFa, there was an increase in BV density and angiogenesis in the cortex following subcutaneous _L_-lactate injections (Fig. 8d–i), resembling aspects of WS effects (Fig. 8k). Importantly, in the S1 cortex injected with shRNA-MCT2 virus, subcutaneous _L_-lactate injections not only increased astrocytic VEGFa (Fig. 8b, c) but also enhanced BV densities (MCT1 + , PDGFRβ + , or Glut1 + ) (Fig. 8d–g), reduced EC cell death (Fig. 8f, g), and stimulated tip EC proliferation (Fig. 8h, i). Additionally, _L_-lactate injections increased _L_-lactate levels in the brain, including the S1 cortex, hippocampus, and cerebellum, which were also unaffected by shRNA-MCT2 virus injection in the S1 cortex (Fig. 8j). These results demonstrate that _L_-lactate injections alleviate angiogenic deficits induced by shRNA-MCT2, supporting the view that _L_-lactate may act as a downstream signaling molecule underlying WS/MCT2-induced angiogenesis (Fig. 8k).

## Discussion

This paper investigates the role of neuronal MCT2, a lactate transporter, in lactate flux and BV formation in the developing mouse barrel cortex. It demonstrates that MCT2 is essential for BV formation under both baseline and whisker-stimulated conditions. Neuronal MCT2 expression increases with whisker stimulation (WS) and decreases when whiskers are plucked. Further in vitro and in vivo studies reveal two key functions of MCT2: (1) facilitating lactate uptake into neurons, which enhances its own expression, creating a positive feedback loop, and (2) elevating lactate levels in the developing S1 cortex. This lactate increase may promote further lactate influx into neurons and astrocytes, stabilizing astrocytic HIF1α and boosting VEGFa expression, which in turn promotes angiogenesis. Based on these findings, we propose a model in Fig. 8k that highlights the pivotal role of neuronal MCT2 in coordinating neuronal activity, lactate flux, astrocytic HIF1α-VEGFa signaling, and BV formation, providing new insights into the interplay between neuro-metabolic coupling and neuron-angiogenesis coupling.

It is well-recognized that neurons typically maintain low lactate levels, resulting in minimal lactate efflux [23, 28]. Consequently, neuronal MCT2 is primarily regarded as responsible for lactate uptake or influx. Several functions of MCT2-mediated lactate uptake in neurons have been reported, including its role in regulating neuronal excitability and survival [65–67]. Here, we provide both in vitro and in vivo evidence that MCT2-mediated lactate influx into neurons enhances its own expression, likely through the promotion of neural activity and immediate early gene (IEG) expression, thereby establishing a feed-forward positive loop (Fig. 2f). Interestingly, a similar phenomenon is observed with MCT1 induction by its substrate, butyrate, in colonic epithelial cells [68]. This suggests that substrate-induced MCT1/2 expression may represent a common mechanism for optimizing the intracellular availability of substrates like lactate and butyrate, which function both as energy sources and metabolic signals influencing various cellular processes.

Beyond its role in mediating lactate influx into neurons, we found that neuronal MCT2 is both necessary and sufficient to promote lactate flux and elevate lactate levels in the S1 cortex in vivo (Figs. 2, 6, and 7). These findings are consistent with reports by Mazuel et al. [69] and Roumes et al. [64], which show that downregulation of neuronal MCT2 in the adult rat barrel cortex abolishes the WS-induced lactate flux and the lactate increase. These observations raise an important question: How does neuronal MCT2 facilitate lactate flux and elevate lactate levels in the S1 cortex? We speculate that lactate uptake by neuronal MCT2 may trigger its communication with MCT1^+^ ECs, potentially enhancing lactate flux from the blood into the developing S1 cortex. This, in turn, could facilitate lactate influx into neurons and astrocytes. While this speculation aligns with the blood-to-brain lactate shuttle (BBLS) hypothesis and offers new insights into neuro-metabolic coupling, it also raises numerous important questions that warrant further investigation in future studies.

On the other hand, we acknowledge reports by Cruz et al. [70] and Wei et al. [71], who provide evidence suggesting that neurons may also serve as a source of lactate. This raises an alternative hypothesis, proposing that neuronal MCT2 could increase lactate levels in the S1 cortex by promoting lactate efflux from developing neurons. However, our in vitro and in vivo data support a role for lactate influx, rather than efflux, in neurons (Fig. 2). Given the higher lactate concentration in the S1 cortex compared to neurons (supplementary Fig. 10), the possibility of neuronal lactate efflux in the developing S1 cortex remains an area for further exploration.

Given the extensive literature supporting the ANLS hypothesis [19–23], where astrocytes are believed to primarily produce lactate for efflux, our findings of extracellular _L_-lactate influx into astrocytes, leading to HIF1a stabilization and elevated VEGFa, seem to contrast with this ANLS model. This raises an important question: Is lactate-influx into astrocytes exclusively observed under non-physiological conditions, or does it also occur in physiological settings?

We propose that lactate-influx into astrocytes could occur under specific physiological conditions, particularly during mouse development, for the following reasons. First, our analysis of lactate’s dose-response on the induction of HIF1α and VEGFa in cultured astrocytes showed that 5 mM lactate was sufficient to trigger these responses. This concentration is comparable to serum lactate levels found under normal physiological conditions or following exercise in mice [46, 72]. Second, we detected higher lactate levels in neonatal S1 cortex lysates compared to those in cultured neurons or astrocytes (supplementary Fig. 10), suggesting the presence of an additional lactate source in the neonatal S1 cortex. Third, serum lactate levels were higher in neonatal (P21) mice compared to young adults (supplementary Fig. 10). In contrast, serum glucose levels, an essential energy source for glycolytic metabolism and lactate production in astrocytes, were lower in neonatal mice (supplementary Fig. 10). This suggests that blood-derived lactate may be a key source of lactate in the neonatal S1 cortex. Fourth, subcutaneous injections of lactate in neonatal mice increased VEGFa expression in astrocytes and promoted blood vessel formation (see Fig. 8d–g), providing in vivo evidence for the BBLS pathway in inducing astrocytic VEGFa and angiogenesis. Finally, our findings are consistent with studies such as those by Morland et al. [52], which show that increased circulating lactate—through exercise or lactate injections in adult mice—leads to heightened VEGFa expression and blood vessel formation in various brain regions.

Based on existing literature and our findings, we propose that lactate efflux and influx may occur in distinct subtypes of astrocytes, at different ages, and under varying behavioral conditions in mice. It is crucial to recognize the heterogeneity of astrocytes; while many likely function as lactate producers through enhanced glycolysis, promoting lactate efflux and supporting the ANLS hypothesis, others may uptake lactate under specific conditions. For instance, as circulating lactate levels fluctuate—being elevated in neonatal mice and increased with exercise—the heightened blood lactate could activate the BBLS pathway, facilitating lactate influx into both astrocytes and neurons. This is particularly relevant during neonatal development when the blood-brain barrier (BBB) is immature or more permeable [73]. While this model aligns with the BBLS hypothesis, it also raises several important questions that warrant further investigation.

Notably, VEGFa expression is predominantly observed in astrocytes, where it is induced by WS in a neuronal MCT2-dependent manner (Fig. 5g, i). This raises questions about how neuronal activity (via WS) or neuronal MCT2 triggers VEGFa expression in astrocytes. Our in vitro and in vivo data suggest that MCT2-mediated lactate flux plays a key role in this process. First, treatment with exogenous _L_-lactate is sufficient to increase astrocytic VEGFa levels both in culture and in vivo (Figs. 6c and 8c). Second, _L_-lactate treatment dose-dependently elevates HIF1α protein levels and decreases hydroxylated HIF1α (HO-HIF1α) in cultured astrocytes (Fig. 6e), indicating that _L_-lactate stabilizes HIF1α by inhibiting its hydroxylation through PHD enzymes, which are critical for HIF1α degradation [54, 55]. Given that HIF1α is a key transcription factor for VEGFa expression, these findings suggest that _L_-lactate likely induces VEGFa by stabilizing HIF1α in astrocytes. Third, _L_-lactate treatment reduces α-ketoglutarate (α-KG) levels in astrocytes (Fig. 6f), which is known to activate PHD enzymatic activity. Thus, the increase in lactate and decrease in α-KG may collectively inhibit PHDs in astrocytes. Fourth, in an in vitro assay, _L_-lactate suppress a-KG activation of HO-HIF1a, stabilizing HIF1a (Fig. 6I, j), in line with a report by Feng et al. [74]. Lastly, the influx of lactate into astrocytes, likely facilitated by MCT1, is essential for the induction of HIF1α-VEGFa signaling, as inhibition of MCT1/2 (AR-C155858) or MCT1/4 (syrosingopine) almost completely abolishes the effects of _L_-lactate in astrocytes (Fig. 6g). Together, these results support a model in which neuronal MCT2-promoted lactate flux into the S1 cortex may facilitate lactate uptake into astrocytes, triggering a metabolic cascade that involves α-KG depletion, suppression of PHD activation, HIF1α stabilization, and subsequent VEGFa expression (Fig. 6p).

While our results support the role of neuronal MCT2 in promoting lactate-induced astrocytic VEGFa expression, it is important to consider findings from Morland et al. [52], who provide evidence that HCAR1 (hydroxycarboxylic acid receptor 1), a lactate receptor, is essential for circulating lactate (administered via subcutaneous injection) to increase VEGFa expression and angiogenesis in the adult brain. Notice that HCAR1 is predominantly expressed in pial fibroblasts and cerebrovascular cells, such as pericytes, and is only weakly expressed in cortical neurons or astrocytes [52, 75]. Our RNA-seq analysis, along with RT-PCR, confirmed the lower expression of HCAR1 in the S1 cortex, and its expression is not induced by WS (Supplementary Fig. 2g). Based on these observations, we propose that while HCAR1 in pial fibroblasts and meningeal vascular pericytes plays a crucial role in circulating lactate-induced VEGFa expression and angiogenesis in the brain, the induction of astrocytic VEGFa and BV formation in response to neural activity (via WS) primarily relies on MCT2-regulated lactate flux within the local brain region, specifically the barrel cortex. This interpretation does not exclude a potential role for HCAR1 in the WS model, which warrants further investigation.

Additionally, our model of lactate-induced HIF1a and VEGFa expression in astrocytes does not rule out other potential mechanisms, such as pH changes, reactive oxygen species (ROS) generation, or lactylation induced by lactate treatment. For instance, elevated intracellular lactate can be converted to lactyl-CoA in the cytoplasm, which then participates in the lactylation of histones and non-histone proteins in the nucleus, influencing the expression of various genes, including VEGFa [25, 76]. We plan to explore these possibilities in future studies.

## Methods

### Animals

C57BL/6 strain of male mice (also called wild type-WT) were randomized into experimental groups used for all the experiments. The NeuroD6-Cre (also called Nex-Cre) mice were kindly provided by Klaus-Armin Nave, as described previously [77]. The Ai3 mice were purchased from The Jackson Laboratory (stock #: 007903, donated by Hongkui Zeng - Allen Institute for Brain Science). The NeuroD6-Cre mice were crossed with Ai3 to generate NeuroD6-Cre;Ai3 mice. All these mouse lines were maintained in C57BL/6 strain background for >6 generations and confirmed by genotyping analysis with PCR. Mice were maintained on a 12 h light–dark cycle with ad libitum access to water and standard food. The investigators were blind to the genotype, treatment, and experimental groups of the animals. All of the experiments with animals were performed with the approval of the Institutional Animal Care and Use Committee of Case Western Reserve University.

### Reagent, antibodies, and viruses

Chemicals were purchased form Sigma-Aldrich unless otherwise indicated, Polyethylenimine (PEI, 765090), Sodium L-lactate (71718), TRITON X-100 (T9284), Tween-20 (P1379), protease inhibitor cocktail (Roche, 11836153001), Neurobasal^TM^-A Medium (Gibco, 2429791), GutaMAXT^M^ (Gibco, 2085650), Opti-MEM^®^ (Gibco, 2193103), AR-C155858 (TOCRIS, 4960), GSK 2837808 A (TOCRIS, 5189), Syrosingopine (ChemFaces, 84-36-6). The following primary antibodies were used: rat monoclonal anti-CD31/PECAM (BD Biosciences, 553369); rabbit polyclonal anti-SLC16A1/MCT1 (Origene, TA321556); rabbit polyclonal anti- SLC16A7/MCT2 (Millipore Sigma, AB3542); rabbit polyclonal anti-SLC16A8/MCT3 (Novus Biologicals, NBP1-59885); mouse monoclonal anti-SMA-647 (Santa Cruz Biotechnology, sc-32251); chicken polyclonal anti-GFP (Aves Labs, GFP-1020); rat monoclonal anti-CD147/BSG (Bio-Red, MCA2283); isolectin GS-IB4, Alexa Fluor™ 647 Conjugate (Invitrogen, 32450); guinea-pig polyclonal anti-NeuN (Millipore Sigma, ABN90); mouse monoclonal anti-β-actin (Cell Signal Technology, 3700S); rat monoclonal anti-mCherry (Invitrogen, M11217); rabbit monoclonal HIF1α (Cell Signal Technology, 3716S); rabbit monoclonal anti-HIF1α-OH (Cell Signal Technology, 3434S); rat monoclonal anti-Podocalyxin (PODXL, R&D system, 192703); rabbit polyclonal anti-Cleaved Casp-3 (Cell Signal Technology, 9661S); guinea-pig polyclonal anti-S100β (Synaptic systems, 287004); rabbit monoclonal anti-ERG-Alexa Fluor™ 647 Conjugate (abcam, ab196149); rabbit polyclonal anti-Ki67(Millipore sigma, Ab9260); rat monoclonal anti-Ki67 (Synaptic Systems, HS-398 117); goat polyclonal anti-PDGFRβ (R&D Systems, AF1042); mouse anti-Glut1 (Millipore sigma, MABS132); mouse monoclonal anti-Oilg2 (EMD Millipore, MABN50); goat polyclonal anti-Iba1 (abcam, ab5076); Polyclonal Goat anti-Egfl7 (R&D Systems, AF3089); rabbit monoclonal anti-HIF2α (Cell Signal Technology, 71565S); rabbit monoclonal anti-VEGFR2 (Cell Signal Technology, 9698S); and rabbit monoclonal anti-pVEGFR2 (Cell Signal Technology, 2478S). The following plasmids were used for AAV virus packaging with AAV-PHP.eB capsid (addgene, #103005) by Nature protocols [78]: AAV-hSyn-GFP (addgene, #50465), AAV-hSyn-nlsGFP/Cre (addgene, #105540), AAV-CAG-eLACCO1.1 (#168789) [63]. The following viruses were purchased: shRNA-MCT2 (m) Lentiviral Particles (Santa Cruz Biotechnology, sc-40116-V), and VEGF shRNA (m) Lentiviral Particles (Santa Cruz Biotechnology, sc-36815-V). The pAAV-hSyn-DIO-mMCT2-mCherry vector (AAV-hSyn-DIO-mouseMCT2-myc-flag-mCherry) and AAV-hSyn-DIO-hMCT2/mCherry vector (AAV-hSyn-DIO-humanMCT2-T2A-myc-flag-mCherry) were engineered from the AAV-DIO-mCherry (Vigene Biosciences) construct. The MCT2 (Origene, RC205361) stop codon was removed and inserted into the backbone by in-Fusion® HD Cloning system (TaKaRa, 639650). The HA-Clover-HIF-1α Wildtype (Addgene, 163365) plasmid was used for transient transfection into HEK293T cells.

### Whisker stimulation

The WS was carried out based on previous studies [11, 39, 79, 80]. In brief, pups were subjected to daily WS for 8 days (from P14 to P21). Each day, pups were gently restrained on the top of an iron metal cylinder, and their right whiskers were manually stimulated for 15 min (3 to 4 Hz) using a Fitchew brush. Mice were euthanized at 90 min after the last stimulation.

### Whisker plucking

Plucking began on postnatal day 7 (P7). For the plucking procedures, pups were initially induced with 5% isoflurane and oxygen as anesthesia, then maintained at 1–3% through the duration of the procedure. All macro-vibrissae (30 whiskers) on the left whisker pad were plucked under a stereoscopic microscope by applying gentle tension to prevent damage to the whisker follicle at the base of the vibrissae [81–83]. Since whisker plucking led to a reduction of vascular network formation in the contralateral, the ipsilateral whiskers were right intact. After the procedure, mice were placed in a pre-warming incubated cage and allowed to recover from the anesthesia. The pups were rubbed with bedding from the home cage before being returned to prevent rejection by their mothers. Plucking was done every 2days until euthanized at P21.

### Bulk RNA-seq analysis

Total RNAs were extracted from the S1 cortex without or with WS (90 min after the last stimulation) using the RNA 6000 Nano Kit (Agilent, Santa Clara, CA) and according to the manufactural protocol. RNA Integrity Number (RIN) was accessed for every sample, and the samples were considered qualified with RIN > 2. These RNA samples were then subjected to RNA-seq analyses by BGI America (Cambridge, MA) using the DNBseq™ platform. First, the reads mapped to rRNA were removed, and raw data was acquired. After filtering low-quality, adaptor-polluted, and high content of unknown base reads from the sequencing reads, clean reads per sample remained on average. Then, clean reads were mapped to the reference genome using HISAT2. The result for each sample suggested the samples were comparable. Comparisons to RNA-seq were normalized to fpkm (fragments per kilobase of exon per million mapped fragments) values. DEseq2 and PossionDis algorithms were used to detect the differential expression genes (DEGs). DEGs were determined with adj.P.value < 0.05 and the Log2 fold change >1. Up- and downregulated gene sets from each time point were then analyzed for enriched Gene Ontology terms using g:Profiler [84] with a Benjamini-Hochberg [85] FDR *p* value correction thresholded at <0.05. Normalized RNA-seq data were provided in Supplementary Table 1. Volcano plot of gene expression profiles were generated by VolcaNoseR, https://goedhart.shinyapps.io/VolcaNoseR/, [86]. Heatmap was generated by GraphPad Prism software. Gene expression profiles were *Z*-transferred. GO enrichment analysis was performed by ShinyGO database, http://bioinformatics.sdstate.edu/go/, [87]. GO terms with *p*-value < 0.05 were defined as significantly enriched. Kyoto Encyclopedia of Genes and Genomes (KEGG) analysis was performed by Gene set enrichment analysis (GSEA) expression profiles in the online public database of WebGestalt, http://www.webgestalt.org/, [88].

### Measurements of lactate, glucose, and α-KG

The _L_-lactate concentration was measured via its oxidation by lactate oxidase into pyruvate and hydrogen peroxide, which was then detected with a highly specific colorimetric probe (Cell Biolabs, Inc., MET-5012). The glucose amount was measured via the presence of NADH, a pro-luciferin reductase substrate, and a proportional amount of recombinant Luciferin light was detected in the samples (Promega, J60121). The alpha ketoglutarate (α-KG) was transaminated with an assay kit for the generation of pyruvate, which is utilized to convert a nearly colorless probe (abcam, ab83431). The fresh tissue sample was homogenized in cold PBS and complete protease inhibitor cocktail (Roche Applied) and centrifuge at 10,000 x g for 10 min at 4 °C. Samples and standards were incubated for 30 min and then read with a standard 96-well colorimetric plate reader (Agilent, Synergy HTX). Samples were compared to a known concentration of lactate, glucose, or α-KG standard.

### Stereotaxic injection of AAV or lentivirus into S1 cortex of neonatal mice

Mice were anesthetized with a mixture of ketamine (10 mg/kg) and xylazine (1 mg/kg). The AAV2/8-hSyn-eGFP, MCT2-shRNA Lentivirus, or scrambled-Lentivirus particles were stereotaxically injected into the primary somatosensory cortex (S1). Coordinates were determined by the mouse brain atlas [89] (distances are mm from bregma): AP: −0.8, ML: ±1.2, DV: −0.4. Mice were returned to their mothers and recovered until whisker stimulation experiments at P14. For AAV-hSyn-DIO-MCT2-mCherry was co-injected with AAV-hSyn-GFP (addgene, #50465) or AAV-hSyn-nlsGFP/Cre (addgene, #105540) viruses. 0.05% trypan blue was used in intracranial injection in the nanoinjector syringe (WPI, Nanoliter 2020 Injector, 300704) held by the stereotaxic manipulator. The pup’s head WAS gently placed between the ear bars of the neonatal frame (KOPF®, 821-921-1721). Stereotaxic coordinates were determined from lambda (distances are mm): AP: 0.8, ML: ±1.5, DV: −0.6 for standard P1 pups. Mice were returned to their mothers and recovered until whisker stimulation experiments at P14.

### RNAscope multiplex In-Situ hybridization

RNAscope assays were carried out as previously described [90]. Specifically, RNAScope Multiplex Fluorescent Reagent v2 (ADC, 323100), the Probe-Mm-Slc16a7/MCT2-C2 (ADC, 509921-C2), Probe-Mm-Slc16a3/MCT3 (ADC, 432931), probe- Mm-Slc1a3/GLAST (ADC, 430781), Probe- Mm-Slc1a3/GLAST-C2 (ADC, 430781-C2), and probe-Mm-VEGFa-O1 (ADC, 21300 A) as well as the HybEZ hybridization oven (321711) were purchased from ACD, Inc., and all the procedures were performed according to the manufactural protocol. Briefly, mice were perfused with 4% PFA at P21. Their brain samples were post-fixed with 4% PFA for 16 h, dehydrated with 15% and 30% sucrose PBS, and then cryosectioned (14 µm thickness). The brain sections were dehydrated in an ethanol series. The sections were treated with hydrogen peroxide to block endogenous peroxidase activity, and then they were incubated in RNA retrieval buffer maintained at a boiling temperature (100 °C to 103 °C) for 15 min. The brain sections were hybridized with the probes, the negative control probe (a gene anti soil bacteria) and the positive control probe (Polr2-RNA polymerase 2) in a 40 °C hybrid oven for 2 h. After washing, the slides were treated with pre-amplifier for 30 min and amplifier for 15 min at 40 °C. Chromogenic detection (AKOYA Biosciences®, NEL810001KT) was performed using Opal^TM^ 520, Opal^TM^ 570 (red), and Opal^TM^ 690 (opera) to label the probe with fluorescence, and immunofluorescent stained samples were imaged under confocal microscope. DAPI was used as the counterstain.

### Tissue processing and Immunofluorescence

Mice with indicated genotypes were anesthetized and perfused transcardially with 4% paraformaldehyde (PFA; pH7.4). The brains were dissociated and post-fixed overnight, then mounted in agarose prior to vibratome sectioning. 40 µm free-floating vibratome sections of brains were collected in PBS, then blocked and permeabilized with blocking solution containing 3% Donkey serum and 0.5% Tween-20 in PBS for 1 h at room temperature and incubated with primary antibodies in blocking solution overnight at 4 °C. The brain sections were then rinsed with 3x PBST buffer. Afterward, samples were incubated with appropriate secondary antibodies at 1:300 (Thermo Fisher Scientific, Alexa Fluor conjugates) for 2 h at room temperature. Brain sections were washed and incubated with DAPI (Thermo Fisher Scientific) to reveal cell nuclei. After washing with PBS, mouse brain sections were mounted for confocal imaging with Zeiss LSM 800 system. Images were also taken with BZX fluorescence microscope.

### Western blotting

Tissues were dissected and homogenized with RIPA lysis buffer containing a complete protease inhibitor cocktail (Roche Applied). Protein was collected by centrifugation at 13000 *g* for 5 min. The protein concentration was measured by the BCA (Pierce Biotechnology) protein kit, and the supernatants were mixed with loading buffer. For western blot analysis, 10% or 12% SDS-PAGE gels were used to separate proteins, and then the proteins were transferred onto nitrocellulose membranes. Afterward, the membranes were incubated with primary antibodies followed by species-specific horseradish-peroxidase-conjugated secondary antibodies (1:5000, Thermo). ECL kit (Pierce, Rockford) was used to visualize the target proteins. All data were normalized by β-actin and analyzed using ImageJ software as described previously [91].

### Primary neuron and astrocyte cultures

The prenatal cortical neuron culture procedure was modified and used based on previous reports [47, 77, 92]. Briefly, cortices were dissected from E17.5 mouse embryos and digested in 0.25% trypsin at 37 °C for 30 min. Dissociated cells were re-suspended in plating media (Neurobasal-A with 2% B27 supplement, 1x Gluta^max^, and 10% FBS) and plated onto poly-L-lysine–coated 8-mm coverslips in 24 well plates or 35 mm dish at a density of 1 × 10^5^ per well for 4 h before replacing the medium with culture medium (Neurobasal-A with 2% B27 supplement and 1x Gluta^max^). To limit glia proliferation, Ara-C (cytarabine) treatment was performed at DIV 2 for 8 to 18 h. Add Ara-C diluted in culture medium (1 μM final dilution). After Ara-C incubation, 80% of the media was replaced with fresh culture medium (400 μL/well of 24 well plates and 1500 μL in a 35 mm dish). Half of the culture medium was changed every 2–3 days.

The neonatal cortical astrocyte culture procedure was modified and used based on previous reports [93]. Briefly, cortices were dissected from P1 ~ P3 mouse pups and digested in 0.25% trypsin at 37 °C for 30 min. Dissociated cell were re-suspended in a single-cell suspension by astrocyte plating medium (Neurobasal-A with 1x N-2 Supplement with One Shot Fetal Bovine Serum; Gibco™, A1261301). Plated cells in poly-L-lysine–coated 35 mm dish at a density of 1 × 10^7^ per well and change the medium 2 days after plating the mixed cortical cells. After 7 to 8 days fresh astrocyte culture medium with continued by shaking the flask at 240 rpm for 6 hr to remove overlaying microglia sit and oligodendrocyte precursor cells (OPC). Discard the supernatant and rinse the remaining confluent astrocyte layer twice with PBS, add 5 ml trypsin-EDTA, and incubate in the CO2 incubator at 37 °C. The experiment was performed 2 days after culture.

### Real time PCR analysis

Total RNA was extracted from the primary somatosensory cortex of 3 mice in each group using TRIzol reagent. Then, purified RNA (5 µg) was used for cDNA synthesis with *GoScript* Reverse Transcription System (Promega). Quantitative real-time PCR was performed by mixing cDNA, gene-specific primers and 2x SYBR® Green ROX^TM^ FAST Mastermix (Qiagen, NE19-1) in the QuantStudio 3 Real-Time PCR System (Applied Biosystems). Each sample was repeated at least 3 times, and the mRNA level was normalized to GAPDH using the 2^-ΔΔCt^ method. Primer sequences are provided in the Supplementary Information.

### Cell counting and average intensity plots

Quantifications were adapted from previous reports [94, 95]. Confocal images (20x objective, 2 um optical sections, and 0.5x zoom) and maximal intensity Z-projections (ten optical sections) were used for quantification. Using the Cell Counter plugin of ImageJ (NIH), we obtained the number of neurons by counting blood vessels maker positive expression or the number of NeuN-positive nuclei co-localizing with DAPI nuclear staining in the Region of Interest (ROI, e.g., primary somatosensory cortex in layer I to VI). Specifically, All quantifications of intensity were done on an ROI that same area in volume (1 × 10^2 ^mm/mm^3^) includes displaying similar numbers of DAPI nuclear stained. Quantification of expression densities was obtained by intensity dividing in each cell type-specific marker (CD31, IB4, or MCT1) with DAPI counter by the area of the ROI. Average intensity plots were done using the Plot Profile function in ImageJ (NIH). We either show average image profile plots in which the intensity value of several adjacent pixels are averaged (10 µm per bin) or average profile plots profiles where values from each layer. For neuronal expression quantified, MCT2-mRNA or MCT-protein expression was randomly selected from the S1 cortex in layer-specific. Microscopic fields (at least 7 fields/layer/animal) were randomly selected from layers I to VI, where these RNA probes or immunoreactivity is well-set in NeuN+ neurons.

### Fiber photometry recording

Mice (C57BL6/J, male, at P18 days, housed at 23 °C with 40–70% humidity) were anesthetized by intraperitoneal (i.p.) injection of the mixture of ketamine (10 mg/kg) and xylazine (1 mg/kg). Holes were drilled on the skull. Glass needles (World Precision Instruments, 504949) were pulled by a puller (Sutter Instruments, MP-1000) and stereotaxically introduced into the left side of the primary somatosensory cortex (S1) (AP, −1.8 mm; ML,  ± 2.25 mm; DV,  −  0.55 mm from the brain surface) according to the mouse brain atlas. 500nL of AAV_B_-CAG-eLACCO1.1 (3 × 10^13^ viral genome (vg) mL^−1^ each) was injected, and needles were settled at the injected places for 10 min to defuse the viral vectors. After the viral injection, a fiber optic cannula (RWD life science) was implanted into the same S1 cortex. A fiber optic cannula was fixed onto the mouse’s skull using dental composite resin. Subsequently, mice were injected with 0.75 mg kg^−1^ atipamezole (i.p) to awaken them and meloxicam (i.p) to prevent pain for five days. At P25, an optical fiber was connected to a fiber optical cannula and fiber photometry, and the recording was carried out using a commercial device (R821, RWD life science) as previously described [96–98]. In brief, 470- and 410-nm laser beams were first launched into the fluorescence cube and then into the optical fibers; the 410-nm laser was used to acquire reference signal and eliminate noise. Circularly permuted green fluorescent protein (cpGFP) control emission fluorescence was collected by the digital signal interface output frequency at 20 Hz. In vivo recordings were carried out in head-fixed mice trained to whisker stimulation. For whisker stimulation, air puffs (50 ms, 29 psi, 5 Hz) were presented to the mouse’s whisker pad for 60 s. The whisker stimulation session was repeated for 5times including 45 s baseline and 100–110 s post-stimulation periods. We derived the value of the photometry signal F as F470/F410, calculating ∆F/F  =  (F – F0)/F0, where F0 is the median of the photometry signal. The average of peak ∆F/F values and the number of events for each mouse were analyzed.

### Statistics

All data are presented as mean ± SD. Analyses were conducted using five or more brain slices from 3–10 mice per group, or cultured cells from more than three independent in vitro experiments. Blood vessel density, length, and branches were quantified by the Vessel Analysis package in imageJ [99] software. Western blot bands were analyzed by ImageJ and normalized using β-actin or α-tubulin. For two independent data comparisons, an unpaired Student’s t-test was used to determine statistical significance. Tukey correction after one-way analysis of variance (ANOVA) was used for multiple group comparisons. All Statistical analyses were performed on GraphPad Prism 9.0. P < 0.05 was defined as a statistical difference.

## Supplementary information

Supplementary information

Uncropped western blot images

Supplementary material

## Data Availability

The accession number of the RNA-seq data reported in this paper was deposited in GEO under the accession number GEO: GSE227622. All data needed to evaluate the conclusions in the paper are present in the paper and/ or the supplementary information.
