# Supplementary material for: Neuronal MCT2 promotes angiogenesis via lactate in the developing mouse neocortex
Source: Cell Death Differ. 2025 Oct 4;33(3):539–56. doi: 10.1038/s41418-025-01581-w (PMC13035839; doi:10.1038/s41418-025-01581-w)

**Supplementary information**

**Neuronal MCT2 promotes angiogenesis via lactate in the developing mouse neocortex**

Daehoon Lee^1,2,#^, Anika Wu^1,#^, Lingling Yao^1^, Shreya Satish^1^, Lin Mei^1^, and Wen-Cheng Xiong^1,2,^*

^1^Department of Neurosciences, School of Medicine, Case Western Reserve University, Cleveland, OH, USA

^2^Louis Stokes Cleveland Veterans Affairs Medical Center, Cleveland, OH, USA

^#^, Equal contributions

*, Corresponding author

[**Wen-Cheng.Xiong@case.edu**](mailto:Wen-Cheng.Xiong@case.edu)

**Fig. S1│Whisker stimulation (WS) induced micro-vessel density and maturation in mouse barrel cortex.**

**a.** A schematic of whisker stimulation (WS) paradigm. The whiskers in one side of mouse snout were daily stimulated manually using a fitchew brush, 15 min per day, for 8 days (P14 to P21). The other side of mouse barrel cortex that received no stimulation (NS) will be used as a control. **b.** Representative images of immunostaining for CD31 (PECAM-1) expression in S1 cortex with or without the stimulation. **c.** Quantification of CD31^+^-BV density in S1 cortex with WS or NS. **d.** Quantification of CD31^+^-BV widths with WS or NS. The data in **c-d** were mean ± SD (n=7 mice per group, ***P* < 0.01, two-way ANOVA and Bonferroni post comparisons test). **e-j.** Immunostaining analysis with indicated antibodies of the S1 cortex. The representative images (**e, g**, and **i**), and the quantiﬁcations (mean ± SD, 5-7 mice per group, ***P* < 0.01, Student's t-test) (**f**, **h**, and **j**) were shown. Scale bar = 50 μm.

**Fig. S2│** **WS induced expressions of lactate transports and metabolism genes in the S1 cortex.**

**a.** Illustration of bulk RNA-seq procedure. **b.** Volcano plot of gene expression profiles compared WS with NS, the red dots indicate up-regulated genes (_Log_FC > 2, *P* < 0.05), the blue dots indicate down-regulated genes (_Log_FC < 2, *P* < 0.05), and gray dots indicate genes without significance (WS: n = 3, NS: n = 3). **c-d.** The red circles revealed up-regulated (**c**), and the blue circles revealed down-regulated gene expression (**d**). The circular shows the differentially expressed genes assigned to KEGG pathways rank. The y-axis indicated an enriched score in biological processes. **e.** GO analysis of DEGs. DEGs enriched in biological processes (BP) were showed. Sankey plots showed WS-induced genes specifically involved in each of the enriched pathways, which was obtained via ConsensusPathDB. The dot plot showed the ratio between gene specific to BP and the total number of genes in each enriched pathway (FDR-adjusted P<0.05). **f.** Heatmap of RNA-seq analysis showing lactate transporter and metabolism gene expression profiles (DEGs, differential expression genes) in the S1 cortex of WT mice with WS or NS (WS: n = 3, NS: n = 3). All data were Z-transformed. Red highlights increased, and blue marks decreased expression. **P* < 0.05 (n = 3 male mice). **g.** RT-qPCR analysis of lactate transporter and metabolism genes’ transcripts in the S1 cortex with WS or NS. Data were normalized by each group's GAPDH *ct*-value and shown as relative expression (fold) (mean ± SD, each n from 3 mice per group, **P* < 0.05, ***P* < 0.01, Student’s t-test). **h-i.** Western blot analysis of homogenates of S1 cortexes with or without WS using indicated antibodies. Representative western blots (**h**) and quantification (**i**) were shown. (Data were normalized with β-actin and shows fold change quantified, 3 mice per group, mean ± SD, **P* < 0.05, ***P* < 0.01, Student’s t-test).

**Fig. S3│Induction of endothelial MCT1-mRNA and MCT3-mRNA expression in layer IV-V by WS.**

**a.** Representative images of co-RNAscope analysis of MCT1-mRNAs (red) with GLAST-mRNAs (green) in S1 cortex (layer IV-V) of WT mice with WS or NS, which were co-immunostaining with CD31 antibody. DAPI was used to counterstain cell nuclei. **b.** Quantification of MCT1-mRNA distributions in indicated cell types (%) with WS or NS. **c**. Quantification of MCT1-mRNAs levels in CD31^+^ cells, GLAST^+^ cells, and CD31^-^GLAST^-^ cells, respectively. The data in (**c**) were shown as mean ± SD (6 mice per group, ***P* < 0.01, Student's t-test). **d.** Quantification of MCT1-mRNA intensity per CD31^+^-BVs in the S1 cortex with WS or NS (Mean ± SD, n= 24 from 6 mice per group, Student's t-test). **e.** Representative images of co-RNAscope analysis of MCT3-mRNAs (red) with GLAST-mRNAs (green) in S1 cortex (layer I-VI) of WT mice with WS or NS, which were co-immunostaining with NeuN or Olig2. Scale bar = 50 μm. **f.** Quantification of MCT3-mRNAs distributions in indicated cell types (%) with WS or NS. **g**. Quantifications of MCT3-mRNA levels in NeuN^+^ cells, GLAST^+^ cells, Olig2^+^ cells, and NeuN^-^GLAST^-^ cells, respectively (Mean ± SD, 4 mice per group, **P* < 0.05, ***P* < 0.01, Student's t-test). Scale bar = 50 μm.

**Fig. S4│WS-induction of MCT2 expression largely in NeuroD6^Cre^ glutamatergic pyramidal neurons.**

**a-b.** Representative images of co-immunostaining analysis of MCT2 with GFP in the S1 cortex (layer IV-V) of NeuroD6Cre;Ai3 mice (a NeuroD6-Cre reporter line with GFP expression) with WS or NS (**a**). Quantification of MCT2^+^GFP^+^and MCT2^+^GFP^-^cells (%) were presented in **b** (5 mice per group). **c-e**. Representative images of co-immunostaining analysis of PV (green) and cFos (red) in S1 cortex (layer IV-V) with WS or NS in WT mice (**c**). The data in c were shown in **d-e** (mean ± SD, n=7 from 3 mice per group, ***P* < 0.01, Student’s t-test). **f-h**. Representative images of co-immunostaining analysis of GFP (green) and cFos (red) in S1 cortex (layer IV-V) with WS or NS in NeuroD6-Cre;Ai3 mice (**f**). Quantifications of data in f were shown in **g-h** (Mean ± SD, n=12 from 5 mice per group, ***P* < 0.01, two-way ANOVA and Bonferroni post comparisons test). **i-j**. Representative images of co-immunostaining of MCT2 (green) and cFos (red) in S1 cortex (layer IV-V) of WT mice with WS or NS (**i**). Correlation analysis of MCT2 with cFos intensity with fitted linear regression (mean ± SD, n =4 mice per group, R^2^ = Correlation coefficient, ***P* < 0.01; linear regression different) was shown (**j**). Scale bar = 50 μm.

**Fig. S5│AR-C155858 inhibition of WS-induced BV formation.**

Mice received daily intraperitoneal injections of AR-C155858 (1 mg/kg) or vehicle, with or without WS, as illustrated in Fig. 1j. **a**. Representative images of co-immunostaining of MCT1 (green) with isolectin GS-IB4 (IB4, red) in barrel cortex layer IV-V with indicated treatments. **b**. Quantiﬁcation of MCT1^+^-BV densities and IB4^+^-BV densities in layers I to VI [mean ± SD, n= 7~14 (area in mm^3^) from 3 mice per group, ***P* < 0.01, two-way ANOVA and Bonferroni post comparisons test] was shown. **c-d**. Body weight (**c**) and tissue mass (**d**) were monitored throughout the study. **e-f**. A representative _L_-lactate assay for plasma lactate levels measured at the end of the AR-C155858 treatment (**e**). The quantifications were shown in (**f**) (mean ± SD, each n from 3 mice per group, **P* < 0.05, ***P* < 0.01, Student’s t-test). **g**-**h**. A representative colorimetric _L_-lactate assay with indicated conditions and tissue lysates (**g**). Quantifications of _L_-lactate levels from S1 cortexes, TA muscle, and liver with vehicle or AR-C155858 were presented in (**h**) (Mean ± SD, 3 mice per group, *, P < 0.05, multiple t-test). **i.** Representative western blots using indicated antibodies of homogenates from S1 cortex with or without WS and with AR-C155858 or vehicle-treatments. **j.** Quantiﬁcation of data in (**i**). Data were normalized by β-actin and α-tubulin in control samples (3 mice per group, mean ± SD, **P* < 0.05, ***P* < 0.01, one-way ANOVA with Tukey's Honestly Significant Difference (HSD) test). The blue star (*) indicates a comparison between with WS or NS. The magenta star (*) indicates a comparison between AR-C155858 and vehicle treatments, while the black star (*) indicates a comparison between each indicated experiment group, respectively. The number of vessels was indicated by the # symbol in the statistic graphs y-axis. Scale bar = 50 μm.

**Fig. S6│** **Generations of viruses for suppression of neuronal MCT2 by lentivirus or exogenous expression of MCT2 by AAV viruses.**

**a-b.** Suppression of neuronal MCT2 expression by lentivirus of MCT2-shRNA. Representative western blots of homogenates from virus-injected S1 cortexes with WS or NS using indicated antibodies (**a**). Quantiﬁcation of data in (**b**). Data were normalized by β-actin and expression level indicates fold change over control in each group (mean ± SD, 2 mice per group, ***P* < 0.01, one-way ANOVA with Tukey's HSD test). **c-d**. Representative images of co-immunostaining analysis of GFP (green), NeuN (cyan), and MCT2 (red) in S1 cortex injected with lentivirus of MCT2-shRNA or scrambled shRNA particles mixed with AAV-hSyn-GFP virus (**c**). Scale bar = 50 μm. Quantiﬁcation of MCT2 protein levels in layers IV-V in each group (**d**) (mean ± SD, 6 mice per group, ***P* < 0.01, Student's t-test). **e-g**. A schematic of the AAV-hSyn-DIO-mMCT2/mCherry-fusion (Cre dependent mouse-MCT2-mCherry expression) and the experimental protocol (**e**). A representative Western bolt analysis of homogenates of S1 cortexes with indicated virus infections and using the indicated antibodies (**f**) and quantification (**g**) were shown. (Data were normalized with GAPDH and shows fold change quantified, 3 mice per group, mean ± SD, **P* < 0.05, ***P* < 0.01, student’s t-test). **h-i.** Representative images of immunostaining analysis of mCherry^+^ neurons in virus infected S1 cortex in NeuroD6^Cre^ mice (**h**, left), co-immunostaining analysis of MCT2 (green) with mChs (red) in the S1 cortex (layer IV-V) with indicated viruses (**h**, right). Quantiﬁcation of data in (**i**). Scale bars = 10 μm. The blue star (*) marks a comparison between WS vs NS, while the black star (*) indicates a comparison between MCT2-shRNA and Scramble-shRNA, respectively.

**Fig. S7│** **Generations of exogenous expression of human-MCT2 by AAV viruses.**

**a-d**. A schematic of the AAV-hSyn-DIO-hMCT2-T2A-mCherry and the experimental protocol (**a**). A representative image of immunostaining analysis of mCherry^+^ neurons in virus infected S1 cortex in NeuroD6^Cre^ mice. DAPI was used as the counterstain (**b**). Western blot analysis of homogenates of S1 cortexes with indicated virus infections and using the indicated antibodies. Representative blots (**c**), and quantification (**d**) were shown. (Data were normalized with GAPDH and shows fold change quantified, 3 mice per group, mean ± SD, **P* < 0.05, ***P* < 0.01, one-way ANOVA test).

**Fig. S8│ Increased pVEGFR signaling by expression of neuronal MCT2 or WS.**

**a-b**. Representative Western blots using indicated antibodies of homogenates from indicated virus-infected S1 cortexes (**a**), and the quantiﬁcation of data in (**b**), which were normalized by GAPDH in each group samples (mean ± SD, 4 mice per group, **P* < 0.05, ***P* < 0.01; Student's t-test). **c-d**. Representative images of co-immunostaining analysis in the barrel cortex with WS or NS using the indicated antibodies (**c**), and the quantification of pVEGFR2^+^-BV densities (**d**) (mean ± SD, n=7 from 3 mice per group, ***P* < 0.01, Student's t-test).

**Fig. S9 │ Requirement of VEGFa for basal and activity-induced angiogenesis.**

**a-b**. Representative images of co-RNAscope analysis of VEGFα-mRNAs (green) with GLAST-mRNA (red) in the presence or absence of WS with shRNA-VEGFa or scrambled lentivirus injections (**a**) and quantifications of data (mean ± SD, n=8 from 3 mice per group, ***P* < 0.01, one-way ANOVA test) (**b**) were shown**. c-i**. Representative images of immunostaining or co-immunostaining using indicated antibodies (**c**, **e**, **g**) and the quantifications (mean ± SD, n=8 from 3 mice per group, ***P* < 0.01, one-way ANOVA test) in (**d**, **f**, **h**, and **i**) were shown. The presence or absence of WS with shRNA-VEGFa or scrambled lentivirus injections were indicated. Scale bars = 50 μm. The blue star (*) denotes a significant difference between WS and NS, while the black star (*) indicates a comparison between each indicated experiment group, respectively. **j**. Summary of neuronal MCT2 and VEGFa functions in promoting BV-endothelial cell proliferation and survival.

**Fig. S10 │ _L_-lactate levels in S1 cortexes, cultured astrocytes and neurons, and serum samples.**

**a.** The _L_-lactate levels in primary cultured neurons, astrocytes, and S1 cortex were shown (Mean ± SD, n = 3~4, **P < 0.01, one-way ANOVA test). **b-d**. A representative colorimetric _L_-lactate assay in serum plasma from 2-month-old or P21 neonatal mice with vehicle or L-lactate injections (s.c., 2g/kg, daily once, P14 to P21). The quantifications of serum _L_-lactate levels by colorimetric assays (**c**) and serum glucose levels by luminescent glucose assay (**d**) were shown (triplet repeat test from 3 mice per group, mean ± SD, ***P* < 0.01; one-way ANOVA test). **e**. Summary of lactate levels in Tissues, primary cultured cells, and serum.


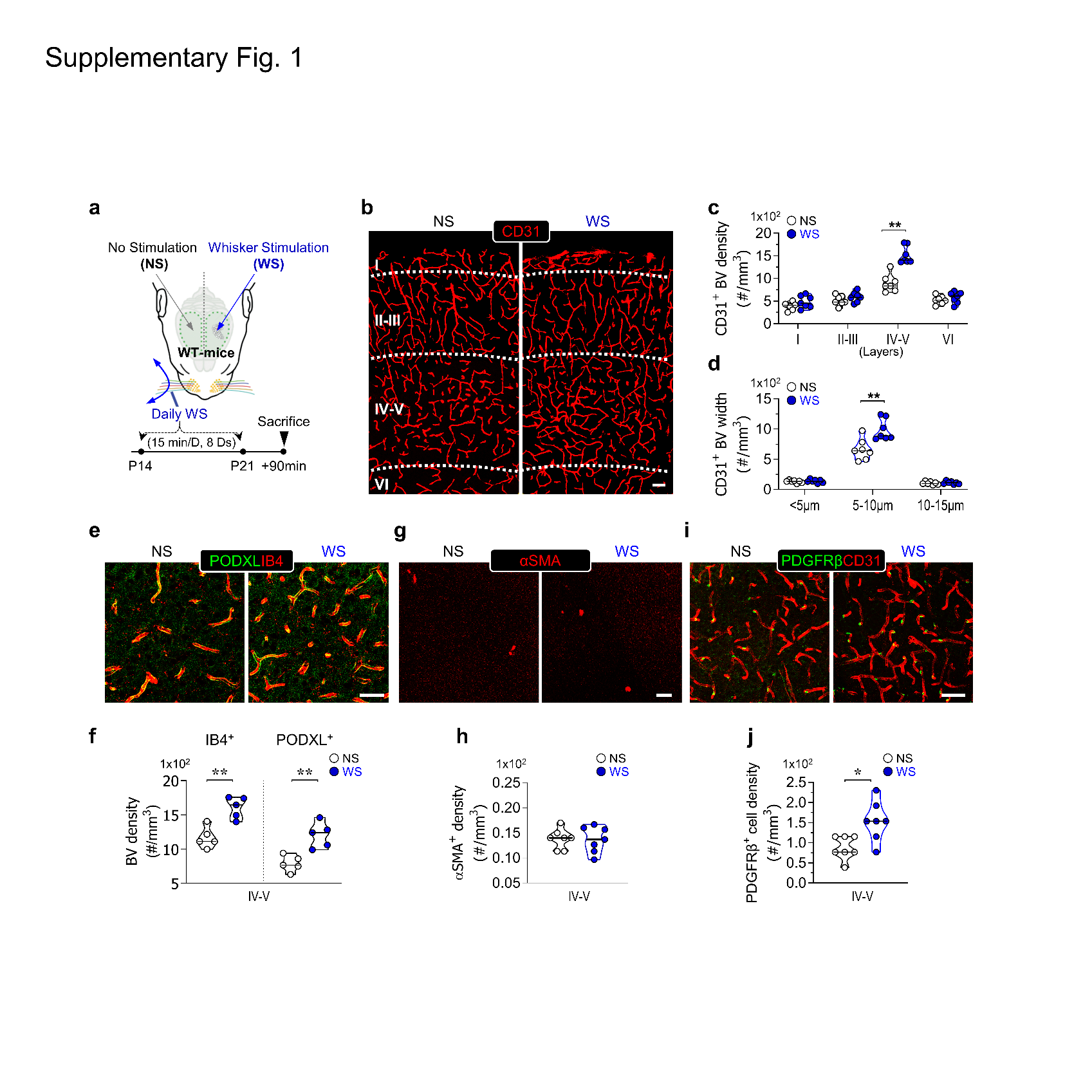


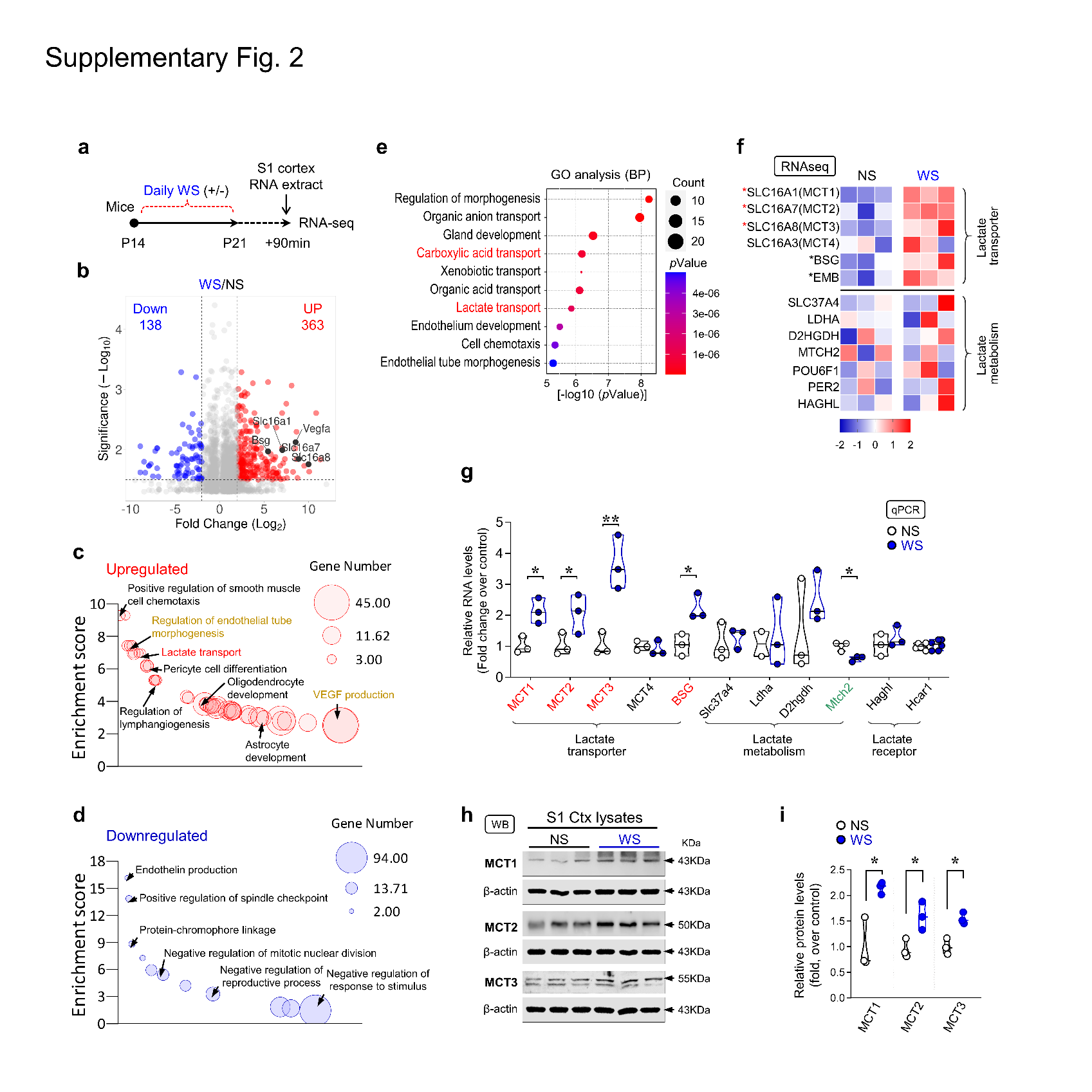


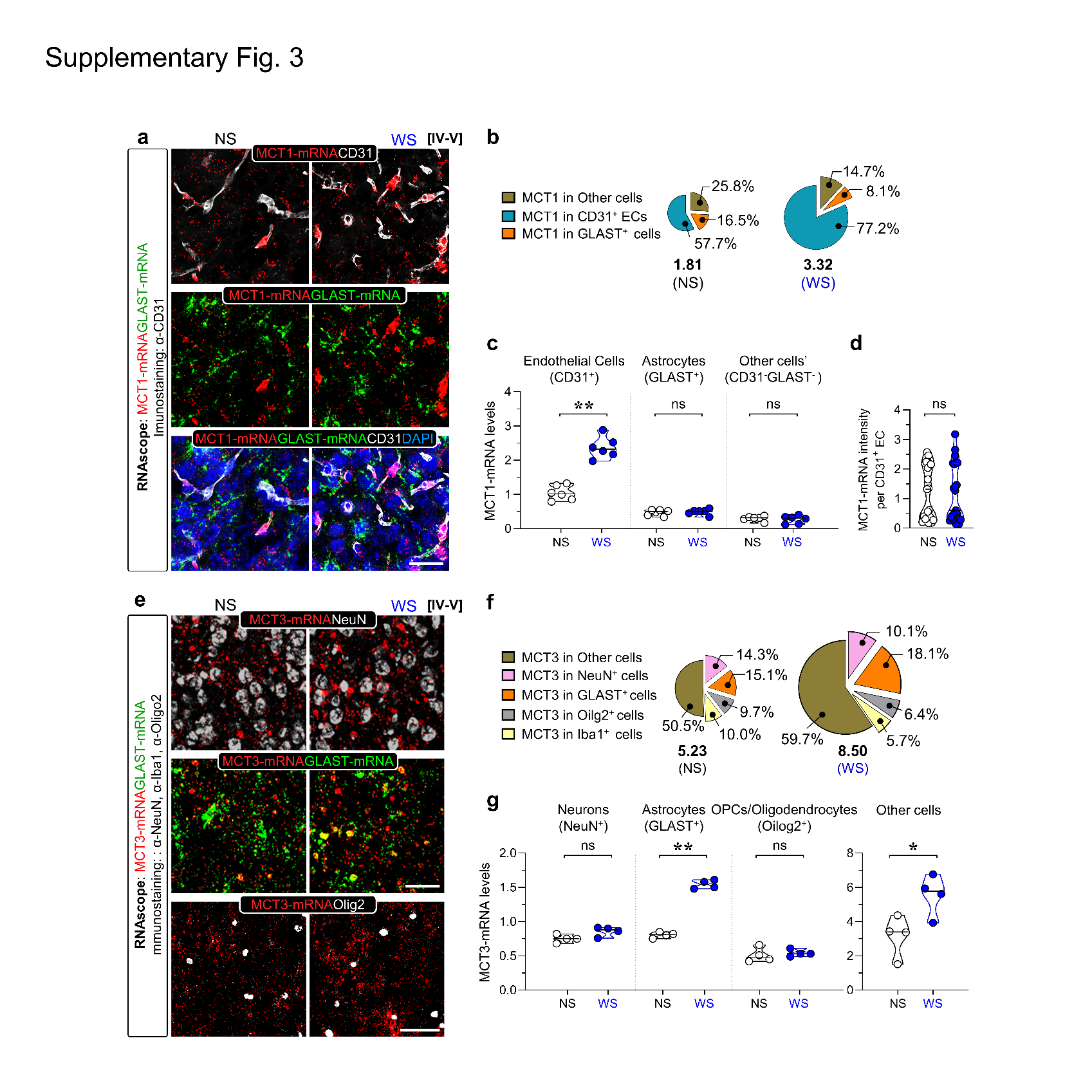


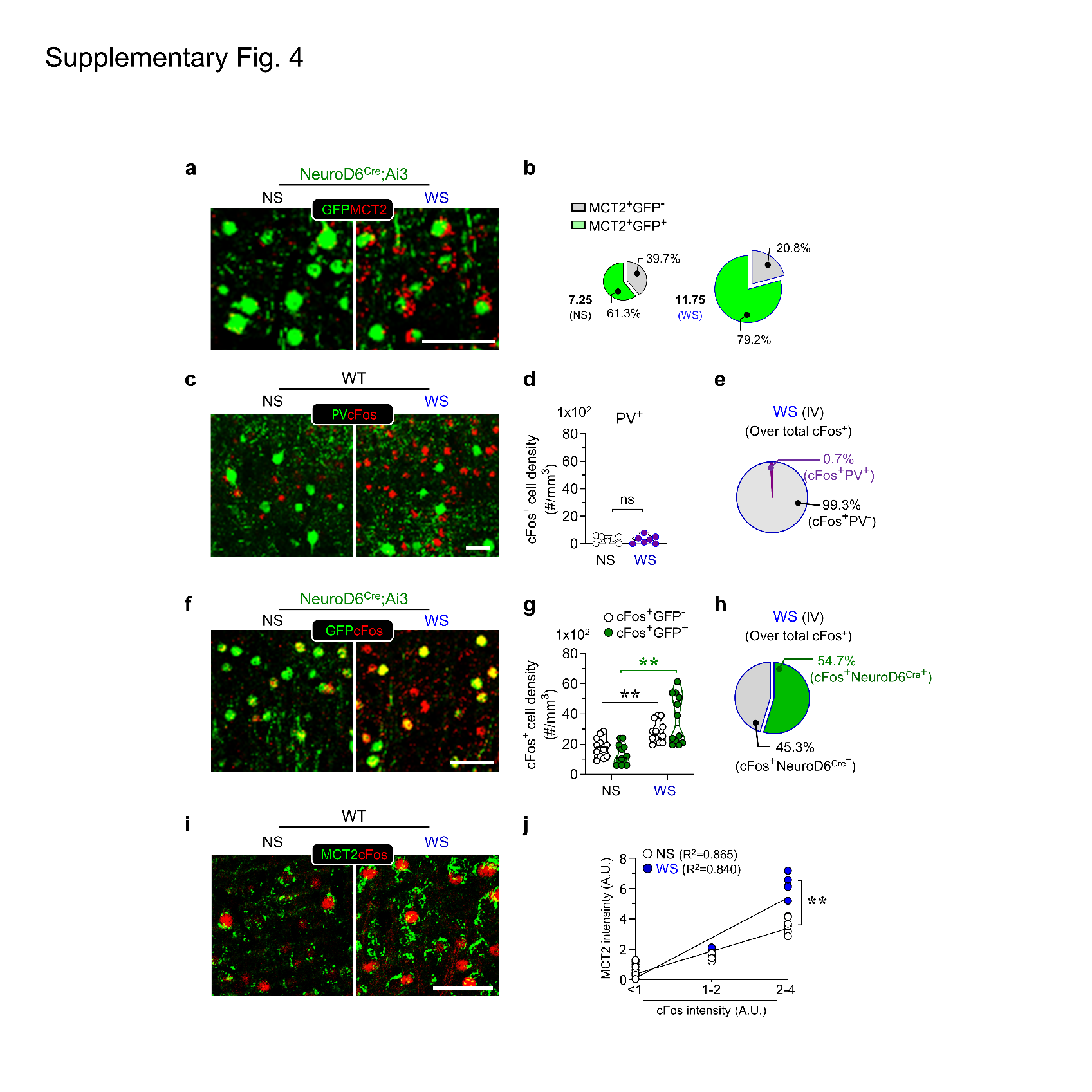


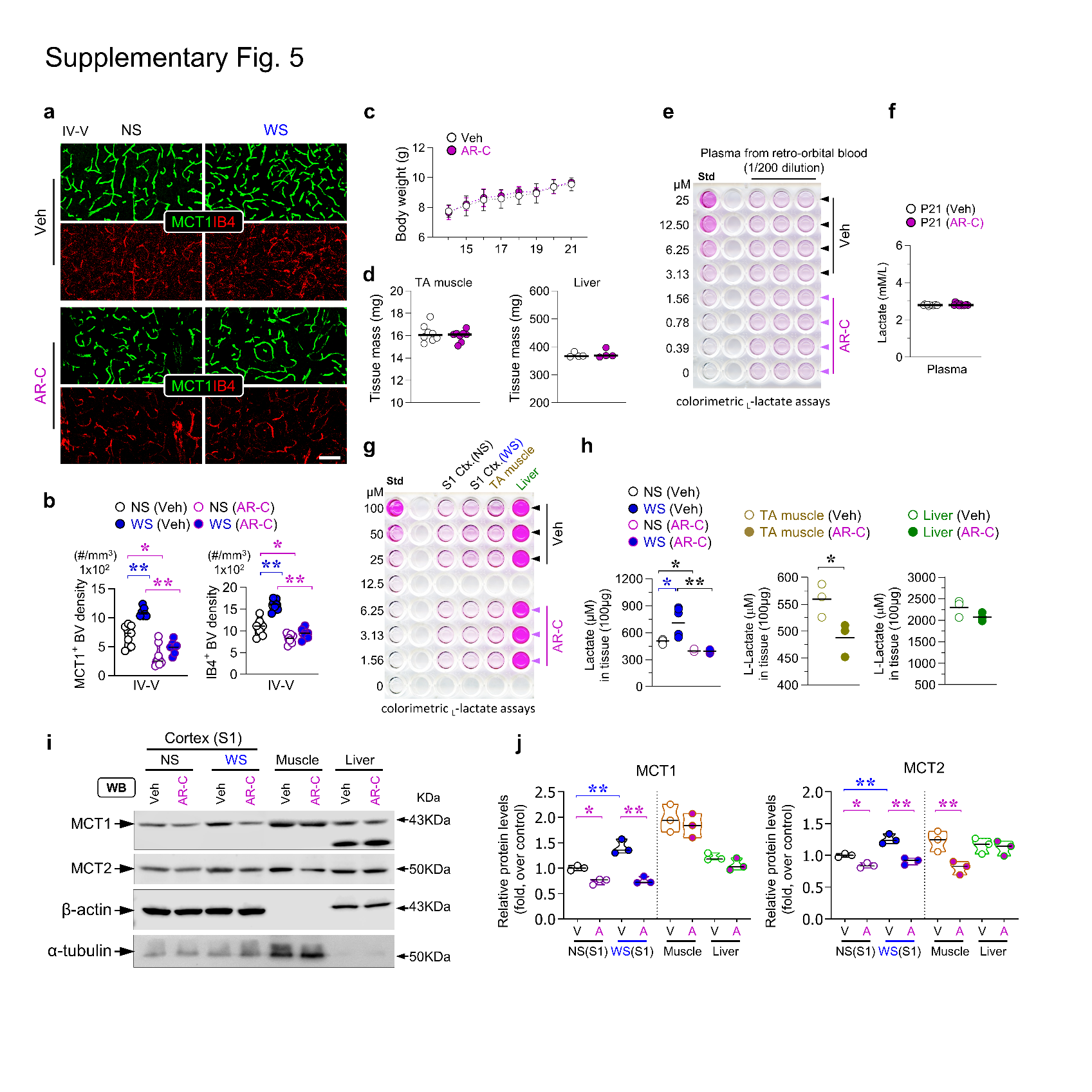


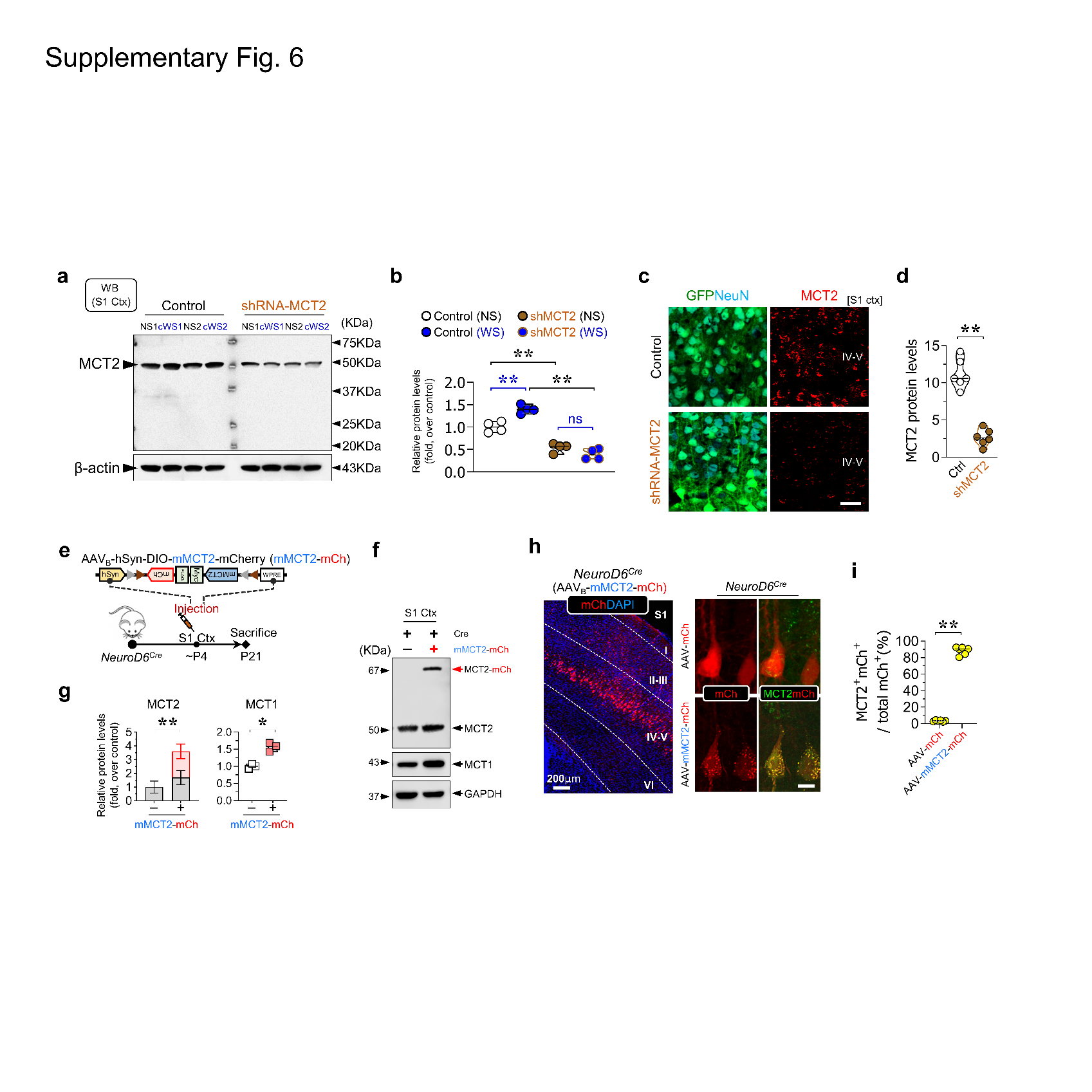


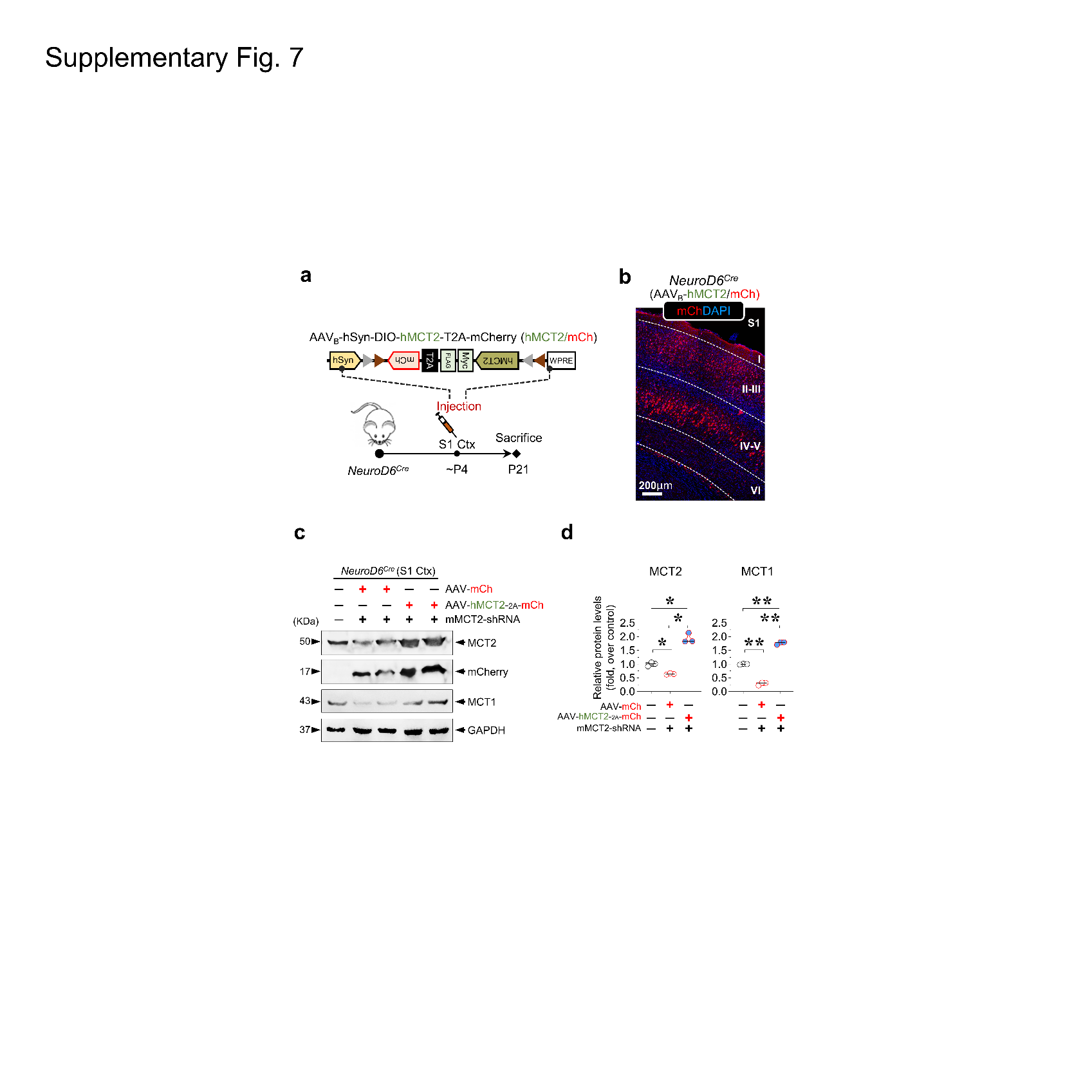


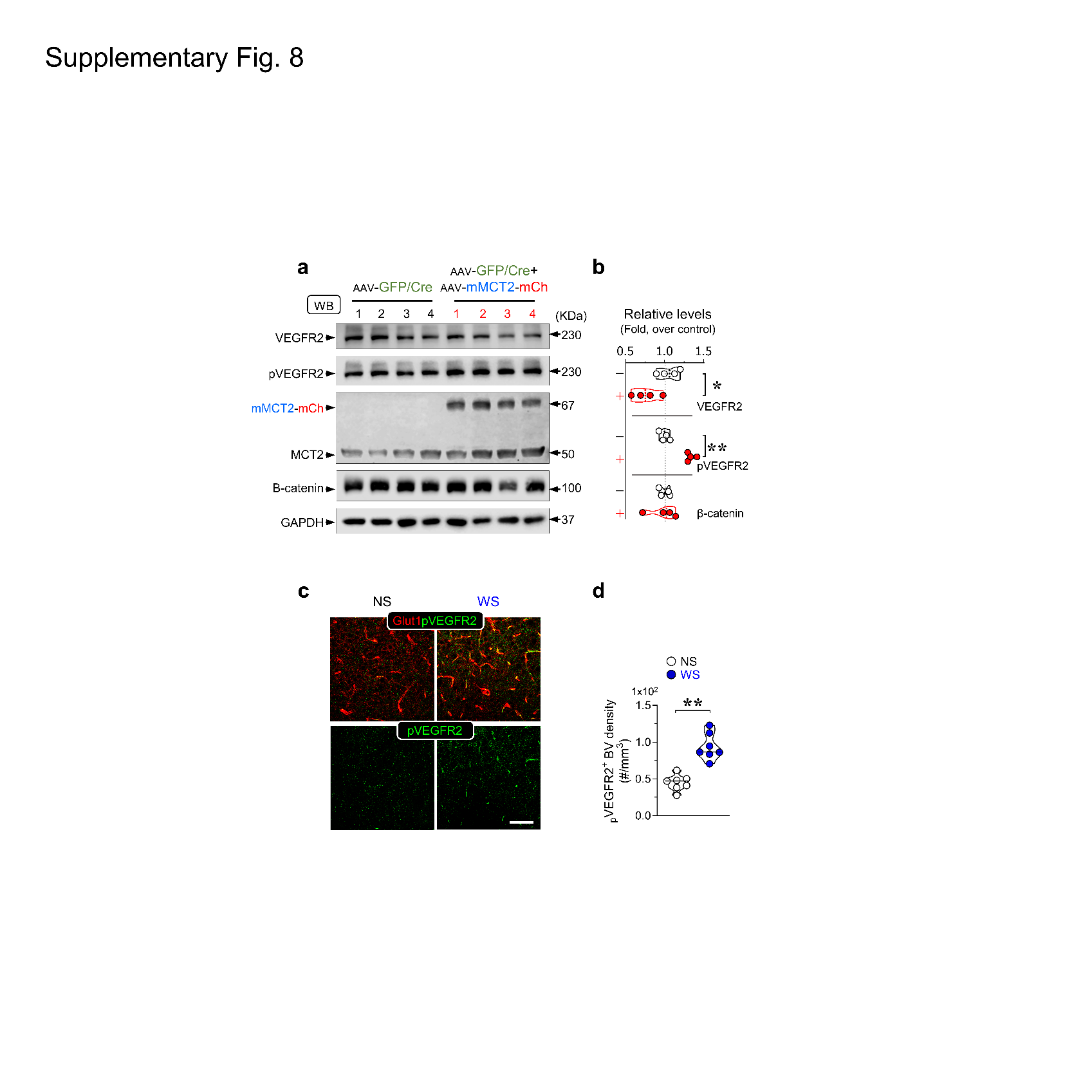


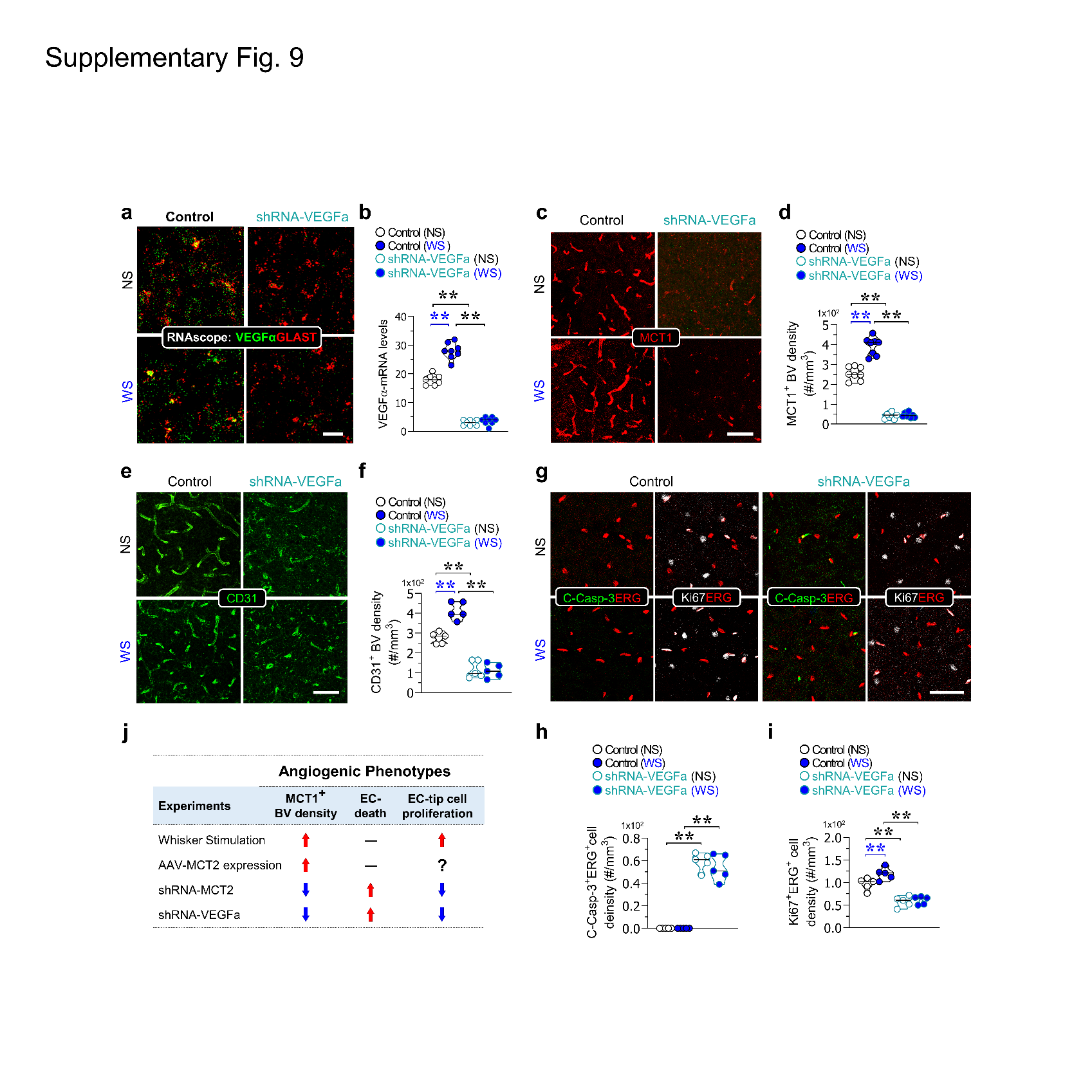


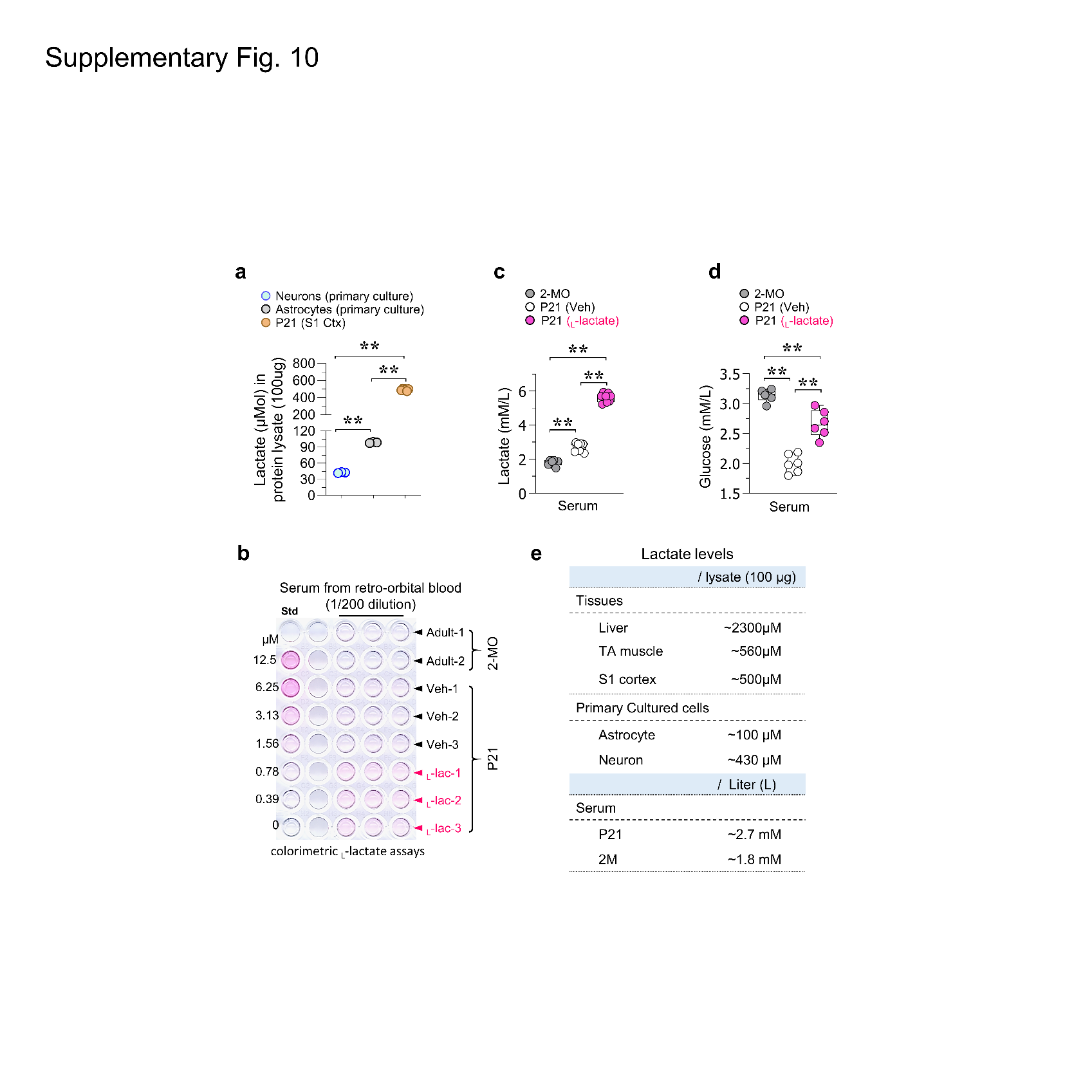

Supplement: Supplementary file 1 — Supplementary information [file 41418_2025_1581_MOESM1_ESM.docx]
