# Supplementary figures and images for: Neuronal MCT2 promotes angiogenesis via lactate in the developing mouse neocortex
Source: Cell Death Differ. 2025 Oct 4;33(3):539–56. doi: 10.1038/s41418-025-01581-w (PMC13035839; doi:10.1038/s41418-025-01581-w)

# Fig. 2b

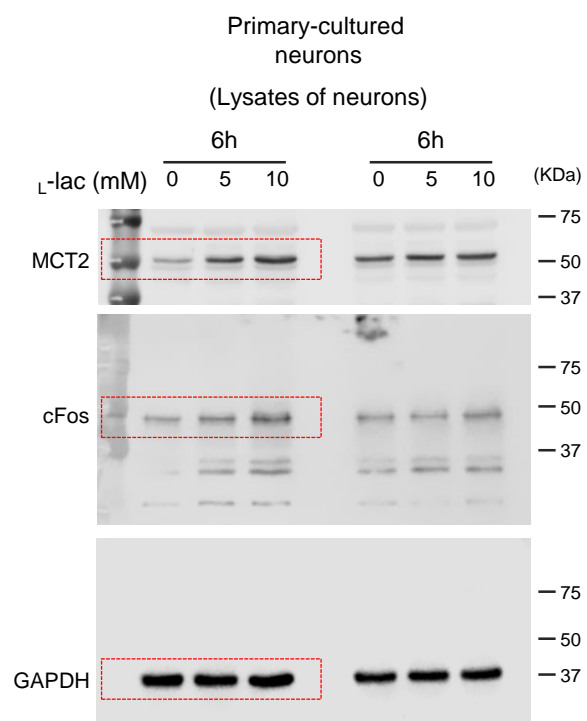

# Fig. 2c

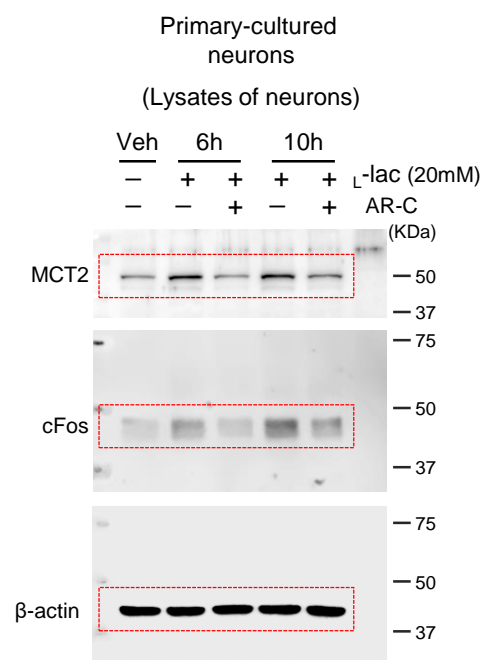

Fig. 6b

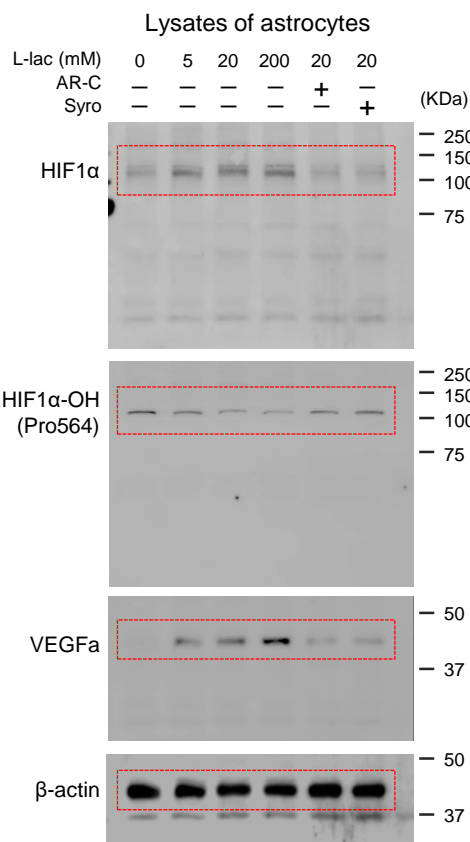

Fig. 6g

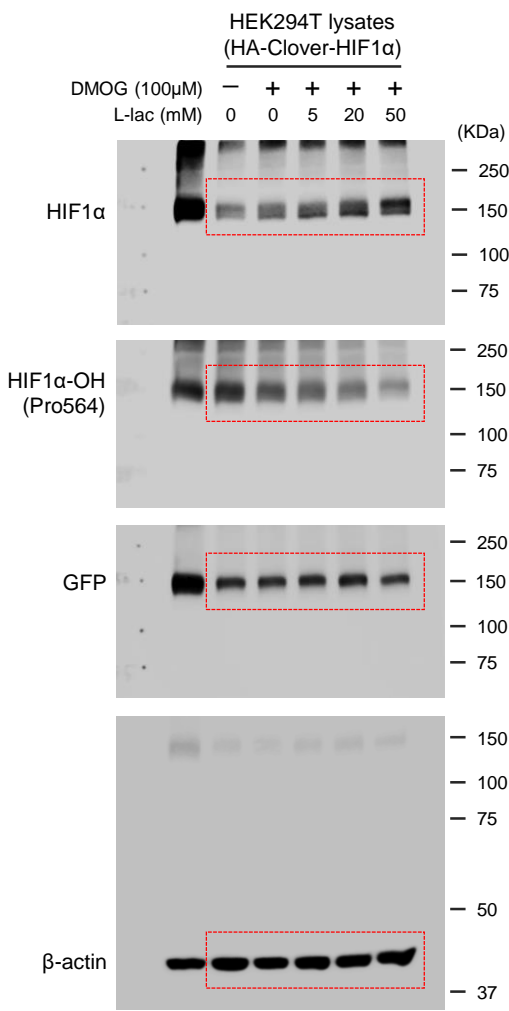

Fig. 6l

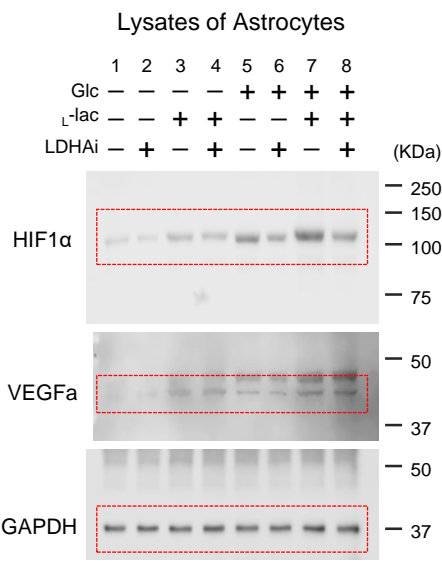

S Fig. 2h

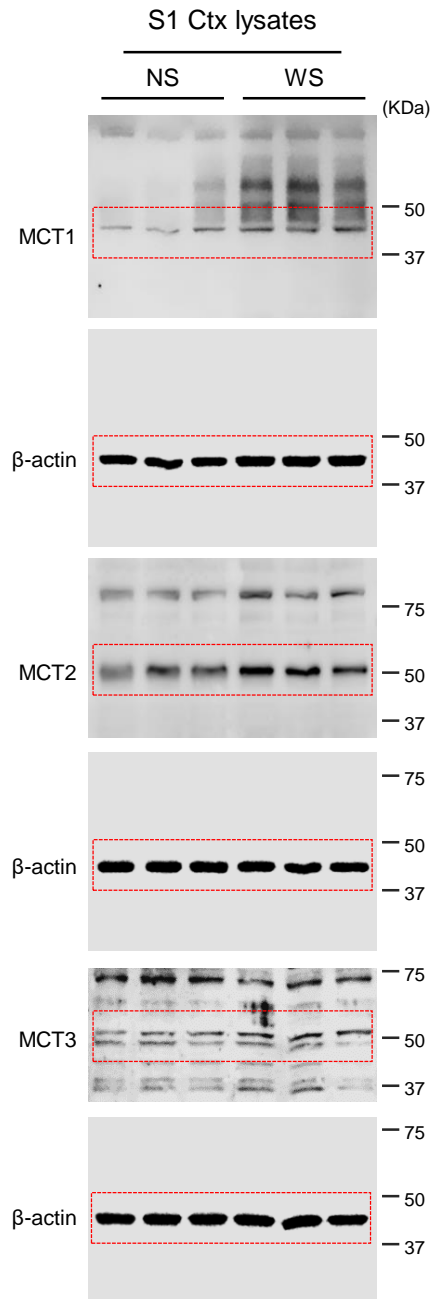

S Fig. 5i

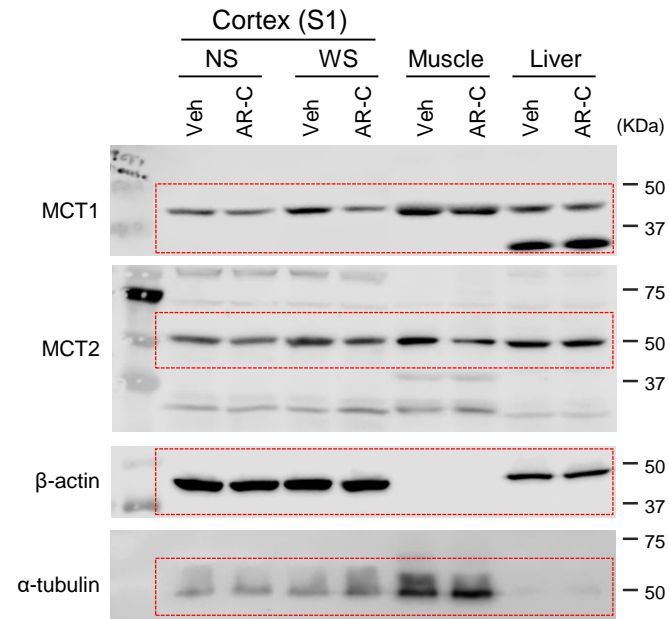

S Fig. 6a

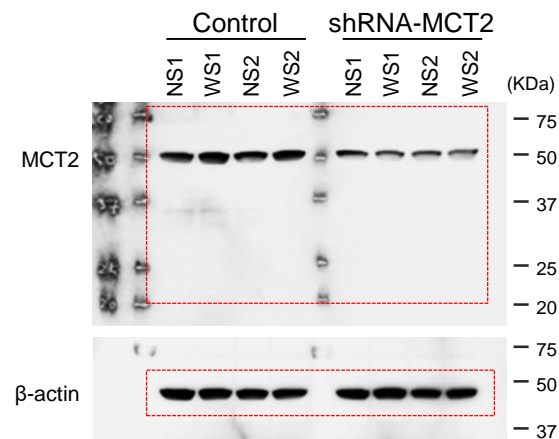

S Fig. 6f

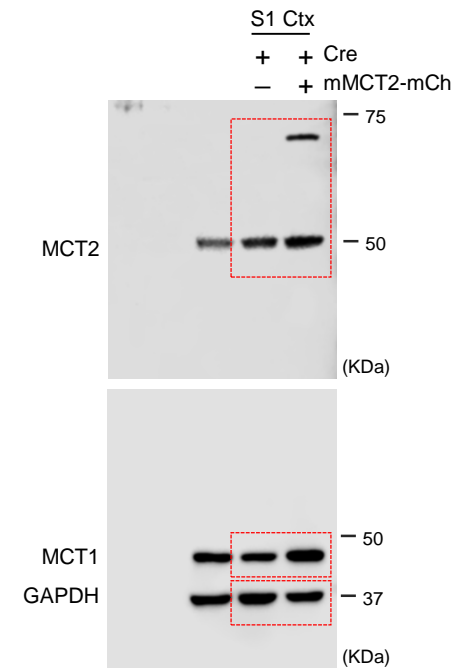

S Fig. 7c

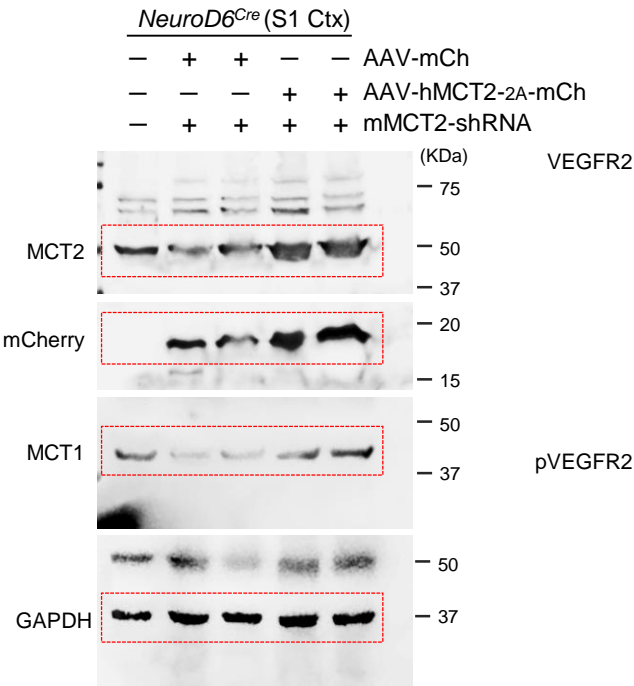

S Fig. 8a

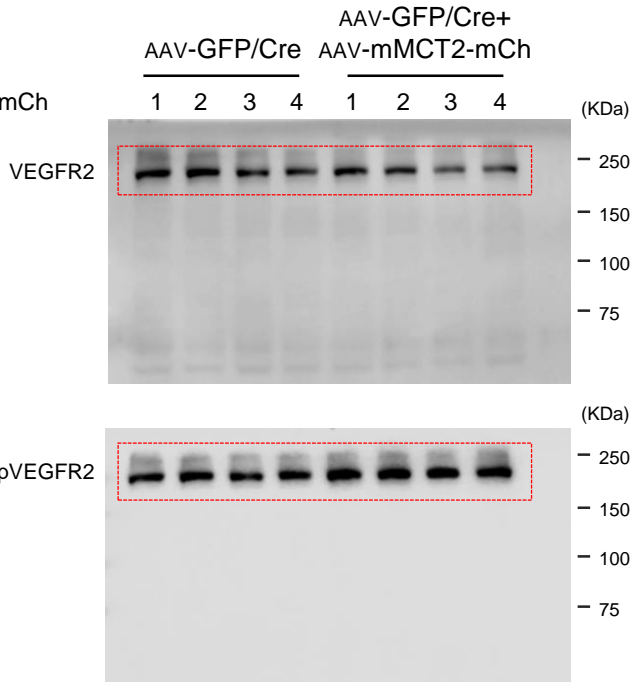

S Fig. 8a

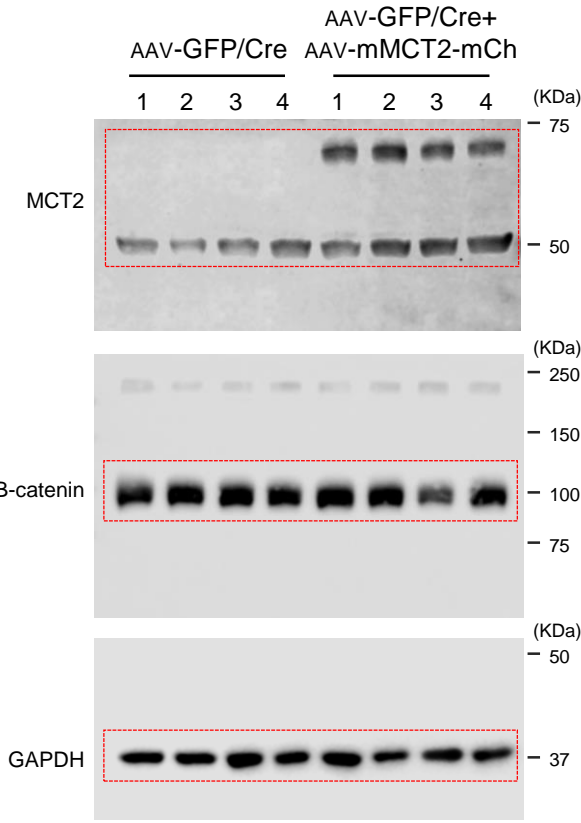

Supplement: Supplementary file 2 — Uncropped western blot images [file 41418_2025_1581_MOESM2_ESM.pdf]
